# Cyclizing Painkillers: Development of Backbone-Cyclic TAPS Analogs

**DOI:** 10.3389/fchem.2020.532577

**Published:** 2020-11-12

**Authors:** Alaa Talhami, Avi Swed, Shmuel Hess, Oded Ovadia, Sarit Greenberg, Adi Schumacher-Klinger, David Rosenthal, Deborah E. Shalev, Mattan Hurevich, Philip Lazarovici, Amnon Hoffman, Chaim Gilon

**Affiliations:** ^1^Department of Organic Chemistry, Institute of Chemistry, The Hebrew University of Jerusalem, Jerusalem, Israel; ^2^School of Pharmacy, Institute for Drug Research, The Hebrew University of Jerusalem, Jerusalem, Israel; ^3^Meytav Technologies Incubator, Kiryat Shmona, Israel; ^4^Department of Pharmaceutical Engineering, Azrieli College of Engineering Jerusalem, Jerusalem, Israel; ^5^Wolfson Centre for Applied Structural Biology, The Hebrew University of Jerusalem, Jerusalem, Israel

**Keywords:** reductive alkylation, backbone cyclization, cycloscan, TAPS, peripheral painkiller

## Abstract

Painkillers are commonly used medications. Native peptide painkillers suffer from various pharmacological disadvantages, while small molecule painkillers like morphine are highly addictive. We present a general approach aimed to use backbone-cyclization to develop a peptidomimetic painkiller. Backbone-cyclization was applied to transform the linear peptide Tyr-Arg-Phe-Sar (TAPS) into an active backbone-cyclic peptide with improved drug properties. We designed and synthesized a focused backbone-cyclic TAPS library with conformational diversity, in which the members of the library have the generic name TAPS c(n-m) where n and m represent the lengths of the alkyl chains on the nitrogens of Gly and Arg, respectively. We used a combined screening approach to evaluate the pharmacological properties and the potency of the TAPS c(n-m) library. We focused on an *in vivo* active compound, **TAPS c(2-6)**, which is metabolically stable and has the potential to become a peripheral painkiller being a full μ opioid receptor functional agonist. To prepare a large quantity of **TAPS c(2-6)**, we optimized the conditions of the on-resin reductive alkylation step to increase the efficiency of its SPPS. NMR was used to determine the solution conformation of the peptide lead **TAPS c(2-6)**.

## Introduction

**Figure 1:**
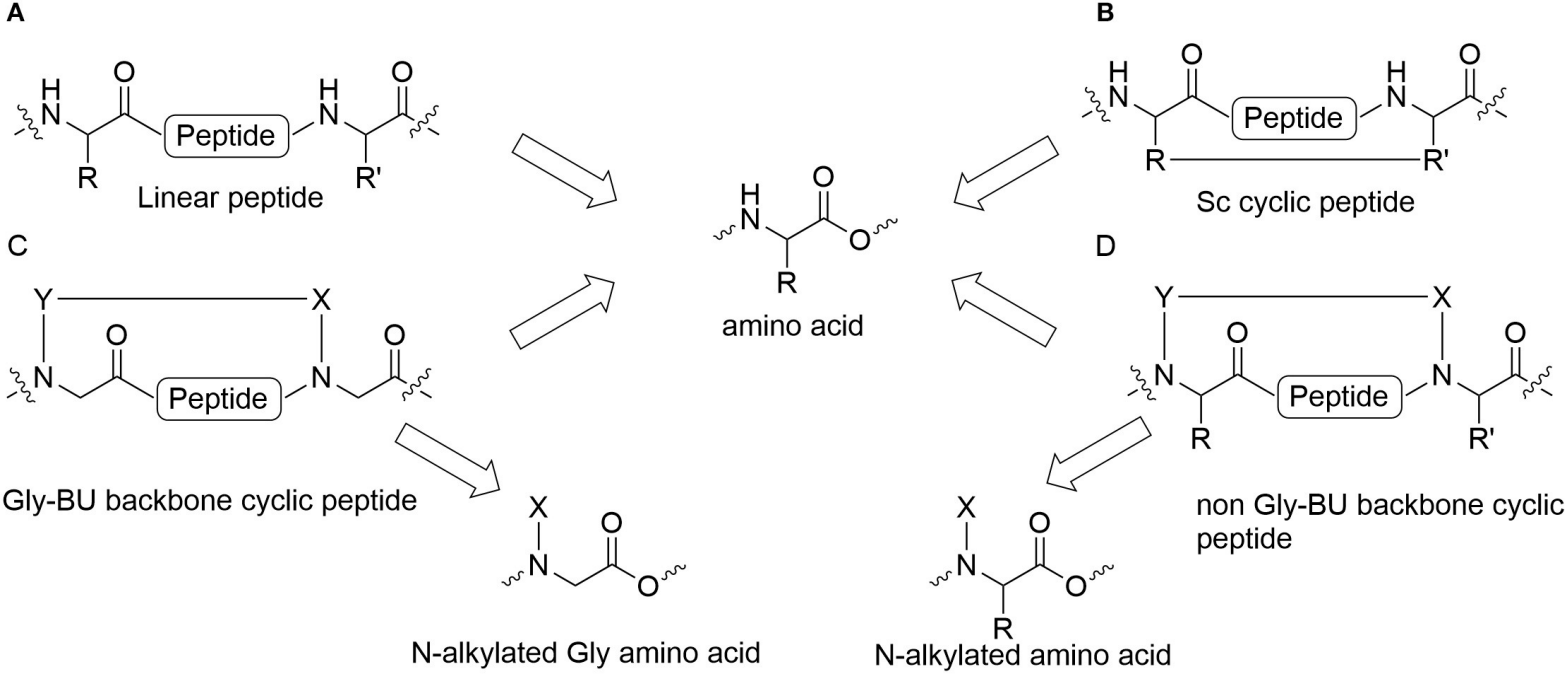
Cyclic peptides and their building blocks. **(A)** Linear peptide; **(B)** Side chain to side chain cyclic peptide; **(C)** Backbone cyclic peptide that employs N-Alkylated Gly amino acids; **(D)** Backbone cyclic peptide that employs non Gly N-Alkylated amino acid.

**Figure 2:**
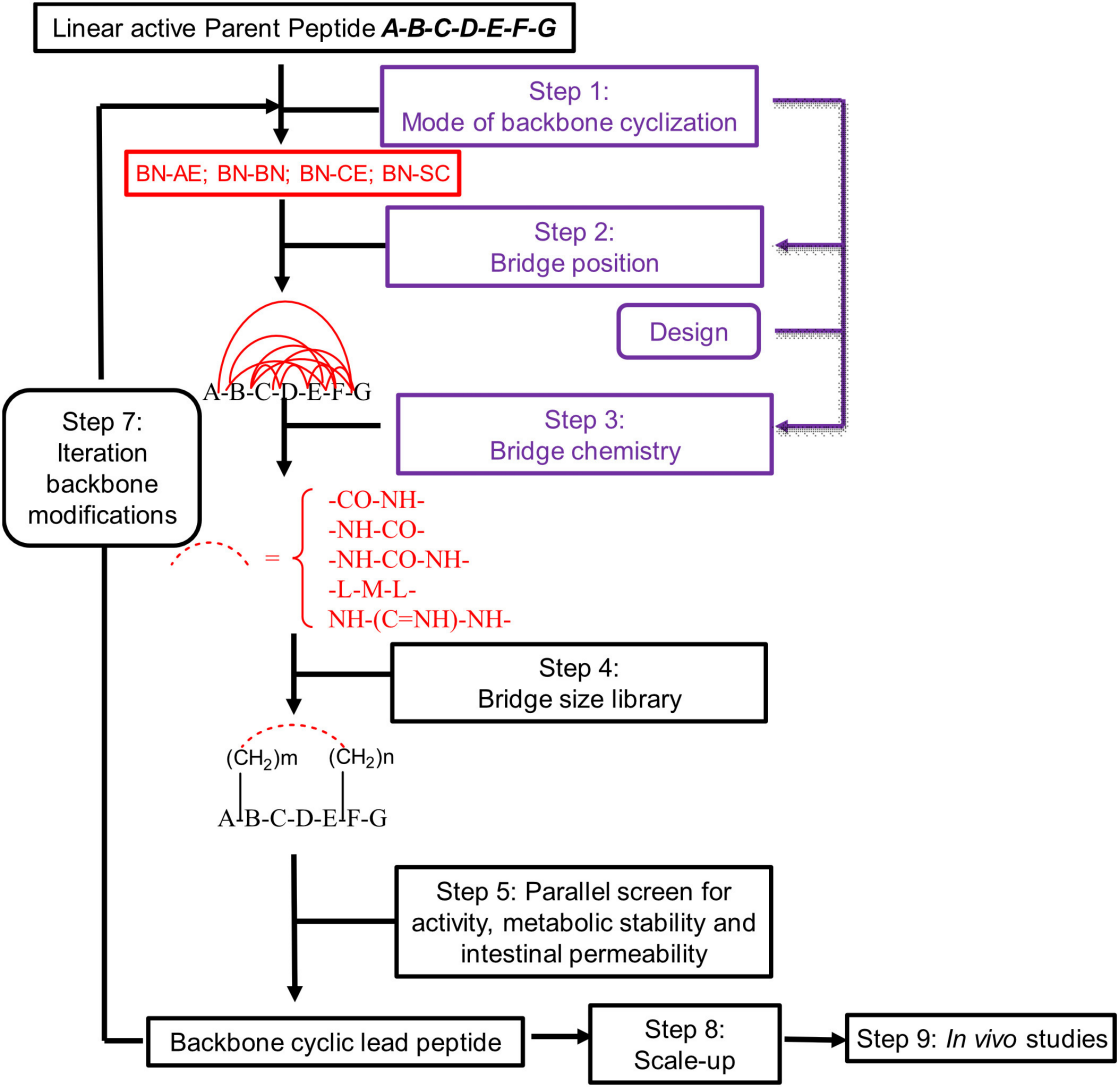
Schematic representation of the steps used for converting an active linear peptide into a backbone-cyclic peptide with drug-like properties. BN-AE, backbone nitrogen to amino end; BN-BN, backbone nitrogen to backbone nitrogen; BN-CE, backbone nitrogen to carboxy end; BN-SC, backbone nitrogen to side chain.

Peptides bearing the pharmacophoric residues necessary for inhibiting protein-protein interactions can induce pharmacologic activity (Pelay-Gimeno et al., 2015). There is a need for new methods for converting pharmacologically active linear peptides (Figure 1A) to drug-leads, which can overcome three main challenges: low metabolic stability, lack of target selectivity and poor oral bioavailability. Cyclization is one of the preferred strategies to achieve some of these goals (Nielsen et al., 2017). To achieve a bioactive conformation of the linear peptide in a cyclic conformation, the optimal cyclic scaffold must be determined empirically. Cyclization is usually performed using the native side chains or termini of the peptide (Figure 1B). Backbone cyclization is a method by which a linear peptide is transformed into a cyclic peptide by skeletal scaffolding. Backbone cyclization was developed as a general technique for the conversion of peptides or the active region in proteins into drug-leads (Fears et al., 2018; Rubin and Qvit, 2018). It is based on cyclization via the α-nitrogens of the peptide backbone (Figures 1C,D). The advantages of backbone cyclization over other cyclization methods are: (i) The ring does not alter the structure of the pharmacophoric side chains and termini of the linear parent peptide; (ii) Backbone cyclization restricts the conformation of the linear peptide thus allowing the design and synthesis of cyclic peptide libraries with a plethora of conformational diversity (e.g., ring position, ring chemistry, size composition, Figure 2); (iii) All the members of the library have the same sequence and differ in their conformation; (iv) Screening a backbone-cyclic library (Cycloscan, Figure 2) enables selection of a backbone-cyclic peptide lead with drug-like properties such as high efficacy, metabolic stability, and selectivity.

Systematic optimization by cycloscan preserves the essential pharmacophores of the linear parent peptide by not using side chains or termini for cyclization, while positioning the pharmacophores in the necessary orientation for binding the target site. Backbone cyclization has proved to increase stability, permeability and potency compared to linear and other cyclic peptides (Hayouka et al., 2010, 2012; Tal-Gan et al., 2011; Hurevich et al., 2013; Rubin et al., 2018).

Establishing a simple and straightforward synthesis of orthogonally protected glycine building units (abbreviated Gly-BU) was an essential step for the facile preparation of backbone-cyclic peptides (Hurevich et al., 2010, 2013). However, using a Gly-BU to replace a non-glycine amino acid from the parent peptide may decrease the potency and selectivity of the resulting backbone-cyclic peptides (Figure 1C). Furthermore, the strategy is not applicable for peptides in which most side chains and/or termini are pharmacophores that are important for binding and activation, or require modifying or extending the active sequence. Contrary to Gly-BUs, synthesizing backbone-cyclic peptides via the α-nitrogen of non-glycine amino acids will preserve all the active pharmacophores (Figure 1D). Developing suitable non-Gly-BUs is crucial for this strategy. Attempts have been made to synthesize such non-glycine building units in solution (Gazal et al., 2001, 2003; Gellerman et al., 2001). However, the need to synthesize each of the BUs in solution limits this approach due to the variety of amino acid functionalities and the large number of optional BUs that have to be prepared. In addition, the synthesis of such orthogonally protected non-Gly-BUs is cumbersome especially for amino acids with functionalized side chains. Reductive alkylation is generally used to allow on-resin N-alkylation of amino acids. However, this strategy is not straightforward since dialkylation can be a dominant outcome for non-optimized protocols.

Backbone cyclic peptide in which the NH is replaced with N-alkyl chain lack some ability to form internal and external H-bonds. Such disadvantage is compensated by the cyclization which can replace the internal H-bonds and the ring itself which usually contains moieties capable of forming additional external H-bonds. The conformational constraint imposed by the N-alkylated based ring can restrict the conformational complementarity of these peptides with the respective receptor and might result in a loss of potency. This implies that while optimal ring size can be suggested, screening a library of backbone cyclic peptides is essential for finding optimal binder.

Opioids are naturally-occurring small peptides that bind and activate opioids receptors in order to manage pain (Gintzler et al., 2019; Romualdi et al., 2019; Zjawiony et al., 2019). The μ opioid receptor (abbreviated MOP) is considered to be the most important pharmacological target for opioid-based treatment (Bodnar, 2019; Manglik, 2020). Endogenic peptides, like endorphins and enkephalins which are biosynthesized from proenkephalin and bind opioid receptors, are produced and secreted by the pro-opiomelanocortin (abbreviated POMC) cells in the brain arcuate nucleus. Their binding to opioid receptors causes closure of the sodium and potassium channels, thereby preventing pain-related signal transduction (Corder et al., 2018; Roques, 2018). Linear enkephalins and endorphins suffer from many pharmacological disadvantages, hence these peptidyl native pain relievers cannot be used as pain relieving drugs (Plein and Rittner, 2018; Sobocinska et al., 2018).

Small molecules like morphine have superior pharmacological properties over linear peptides and are the most widely used painkillers. However, long-term use of these drugs results in various adverse effects such as severe addiction, respiratory depression and loss of effectivity due to acquired tolerance.

One of the most viable alternatives to small molecule painkillers is to develop native endogenic peptide pain relievers with enhanced drug-like properties. Various strategies have been developed over the years to produce stable enkephalin and endorphin mimetics (Karad et al., 2017; Altman et al., 2018; Harris et al., 2019). These short peptides contained all the essential pharmacophores of the parent peptide that proved crucial for bioactivity. Many of these attempts used cyclization as the method of choice to metabolically stabilize the peptides while preserving their natural activity. Cyclization strategies such as side chain-to-side chain cyclization as well as head-to-tail cyclization and ring-closing olefin metathesis were used to synthesize opioid cyclic peptide analogs (Li et al., 2016; Remesic et al., 2016; Nielsen et al., 2017; Stefanucci et al., 2017; Bedini and Spampinato, 2018; Machelska and Celik, 2018; Rubin and Qvit, 2018; Weltrowska et al., 2019; Jing and Jin, 2020; Shinbara et al., 2020). Screening libraries of these cyclic peptides produced biologically active compounds with significantly improved chemical and biological stability, but also proved that there is a major effect of the cyclization method and ring size on the biological activity. Notably, although cyclic peptides with various ring sizes were active, those between 14 and 18 atoms were especially active as they probably allow enough flexibility to adopt the receptor binding conformation (Deschamps et al., 1998; Perlikowska et al., 2016; Weltrowska et al., 2019). N-methylation further restricted the conformation and improved the structural stability of cyclic opioids. This strategy was highly beneficial for allowing an induced fit binding mechanism to the receptor while imposing constraints (Deschamps et al., 1998; Adamska-Bartlomiejczyk et al., 2017). Nevertheless, some of these peptides had many shortcomings that resulted in a partial loss of activity relative to the native peptides. Although cyclization and N-methylation were combined to provide active opioids with good pharmacological properties, the synthesis of these peptides required the use of side chains for cyclization and additional N-methylated positions. N-alkylated amino acids with a functional alkyl chain that can be used for cyclization, provide a viable alternative since they combine both the methylation and the cyclization functions. In addition, by using the functional N-alkyl chain for cyclization, the bioactive side chains are not modified or used for cyclization.

The dermorphin analog tetrapeptide Tyr-D-Arg-Phe-Sar (TAPS) is composed of a single amino acid linker, D-arginine, between the tyrosine and the phenylalanine, and sarcosine (N-Methyl Glycine, Sar) at the C-terminus. TAPS has been shown to be specifically selective for the μ_1_ subtype of the μ opioid receptor (MOP). Structure activity relationship studies have shown that D-Arg^2^ and Sar^4^ enhance activity compared to D-Ala^2^ and Gly^4^, respectively (Broccardo et al., 1981; Glaser et al., 1981; Sasaki et al., 1984, 1985a,b; Suzuki et al., 1985; Sato et al., 1987; Erspamer, 1992; Vardy et al., 2015).

With this background, we developed a short backbone-cyclic peptide opioid analog based on the TAPS sequence that combined both selective agonistic activity toward the μ opioid receptor and metabolic stability. We designed and synthesized stable and active cyclic peptide painkillers based on the shortest possible linear peptide analogs. A novel solid phase peptide synthesis (abbreviated SPPS) strategy of on-resin reductive alkylation was optimized to produce non-Gly building blocks, which facilitate the accessibility of backbone-cyclic peptide analogs. The activity of the TAPS c(n-m) analogs library members as pain relievers was evaluated using *in vitro* and *in vivo* studies and provided some guidelines for finding a future lead.

## Results and Discussion

### Design of the Backbone TAPS c(n-m) Focused Library

**Figure 3:**
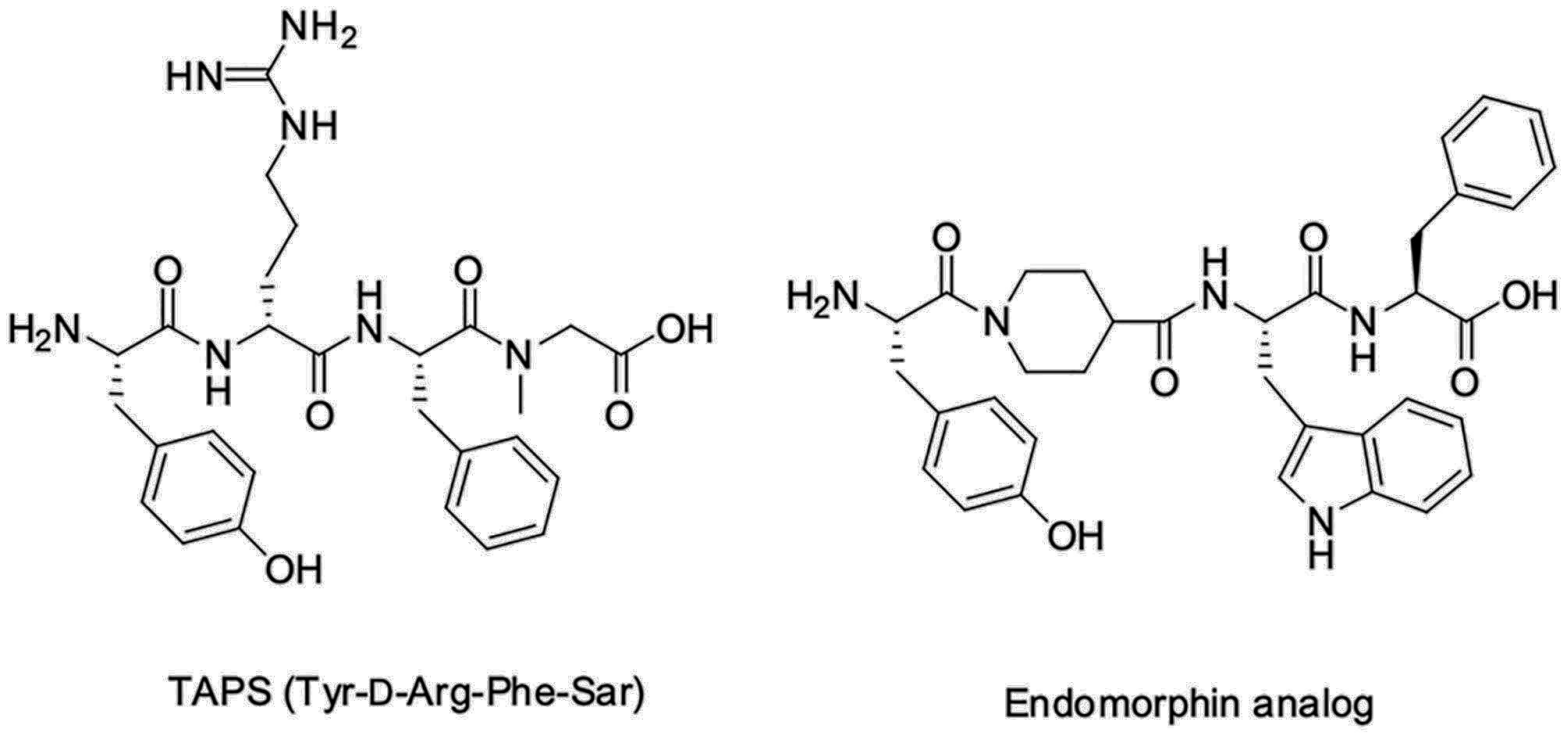
TAPS and β-Endorphin linear tetrapeptide analogs.

We decided to design backbone cyclic mimetics of the TAPS peptide. Applying the backbone cyclization strategy for TAPS was promising but challenging. The design of the focused backbone-cyclic TAPS peptide library was performed according to the general scheme shown in Figure 2. We determined the amide nitrogens of the linear parent peptide which are suitable anchors for backbone-to-backbone cyclization. Two previously published short analgesic peptide analogs, TAPS (Sasaki et al., 1984, 1985a,b; Suzuki et al., 1985) and a β-Endorphin analog (Cardillo et al., 2000, 2002; Gentilucci, 2004; Bedini and Spampinato, 2018), were identified as potential parent peptides (Figure 3). Both peptides contained the conserved amino acids namely N-terminal tyrosine and a phenylalanine at position 3, differing in the size and structure of the spacer between these two amino acids.

**Figure 4:**
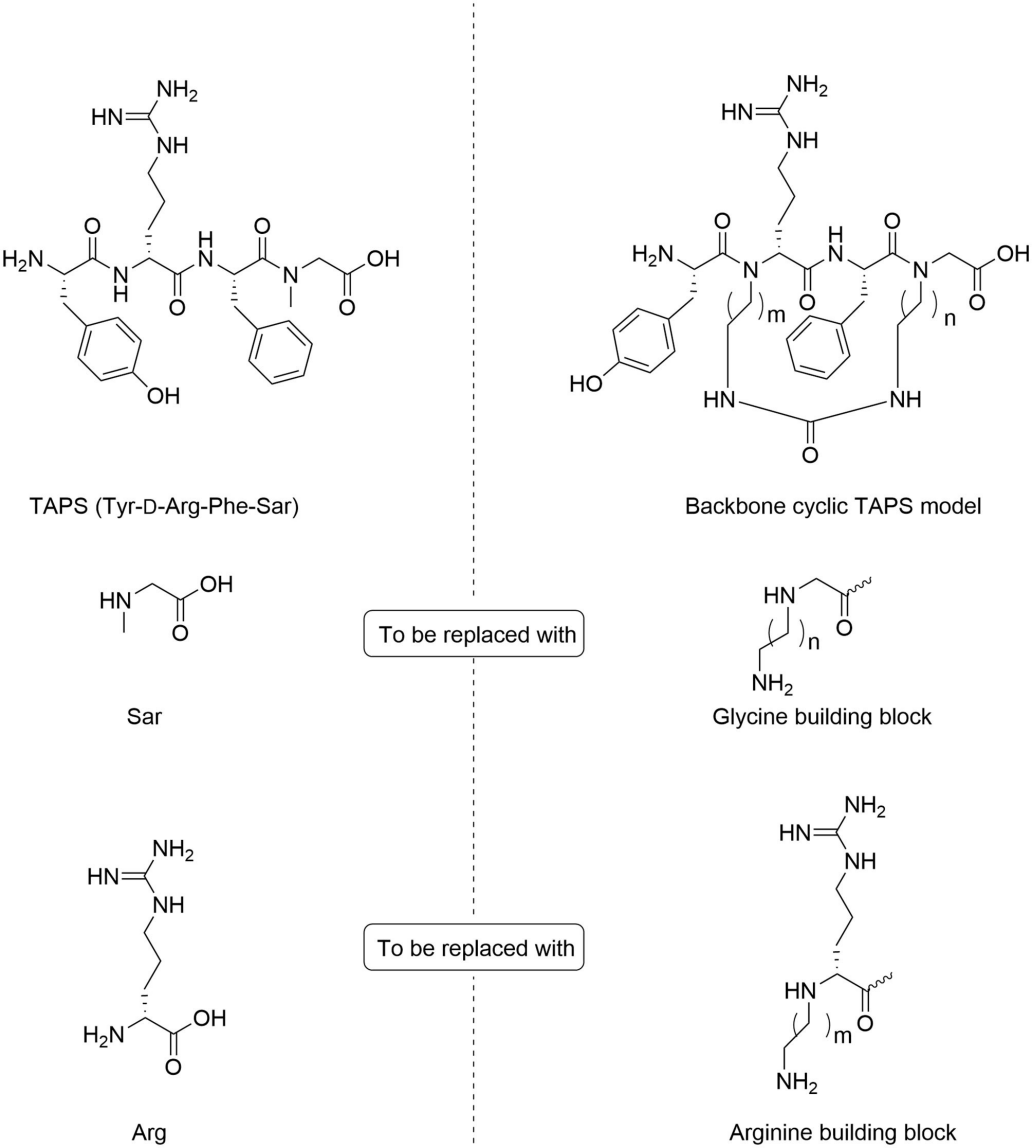
Design of backbone-cyclic TAPS analog. The TAPS linear peptide and building block **(left)**. The backbone-cyclic TAPS analog and building blocks **(right)**.

In TAPS, both Tyr and Phe side chains and the amino terminus are crucial for biological activity (Deschamps et al., 1998). This implies that the cyclization strategy should entail minimal modification of the parent sequence (Figure 3). We choose to synthesize backbone cyclic TAPS analogs that utilize two epsilon-amino alkyl amino acids and are cyclized through urea bridge. N-alkylation allows us to cyclize the peptide without replacing the active functional groups and the urea bridge enabled us to use a set of building blocks with terminal amino functional groups for cyclization. These features have two major advantages: (i) the synthesis of the library is easier since it relies on the same synthetic steps and its amine-protected groups can be removed simultaneously, (ii) both N-alkylation and urea cyclization contribute to the stabilization of the peptide toward metabolic degradation (Tal-Gan et al., 2011; Rubin et al., 2018). We decided to replace the sarcosine of the TAPS parent peptide with an amine functional alkylated glycine building unit for backbone cyclization. Since sarcosine already has an N-alkylated nitrogen, replacing it with a glycine building unit was considered as an anchor for cyclization while preserving the original *N*-alkyl character. Since the sequence of the other three amino acids is crucial for TAPS potency, they could not be replaced by additional Gly-BUs to enable cyclization (Figure 4).

Analyzing the SAR of the endogenous peptide ligand, endomorphin-2 (Tyr-Pro-Phe-Phe-NH_2_), we noticed that the spacer amino acid between Tyr and Phe, proline, is naturally N-alkylated. Therefore, to keep both the integrity of the pharmacophores and still enable the cyclization, we decided to alkylate the α-nitrogen of the TAPS D-Arginine with various epsilon-amino alkyl chains and use it as the second anchor for cyclization (Gazal et al., 2001; Hurevich et al., 2010).

### Synthesis of the Backbone-Cyclic TAPS c(n-m) Peptide Library

**Figure 5:**
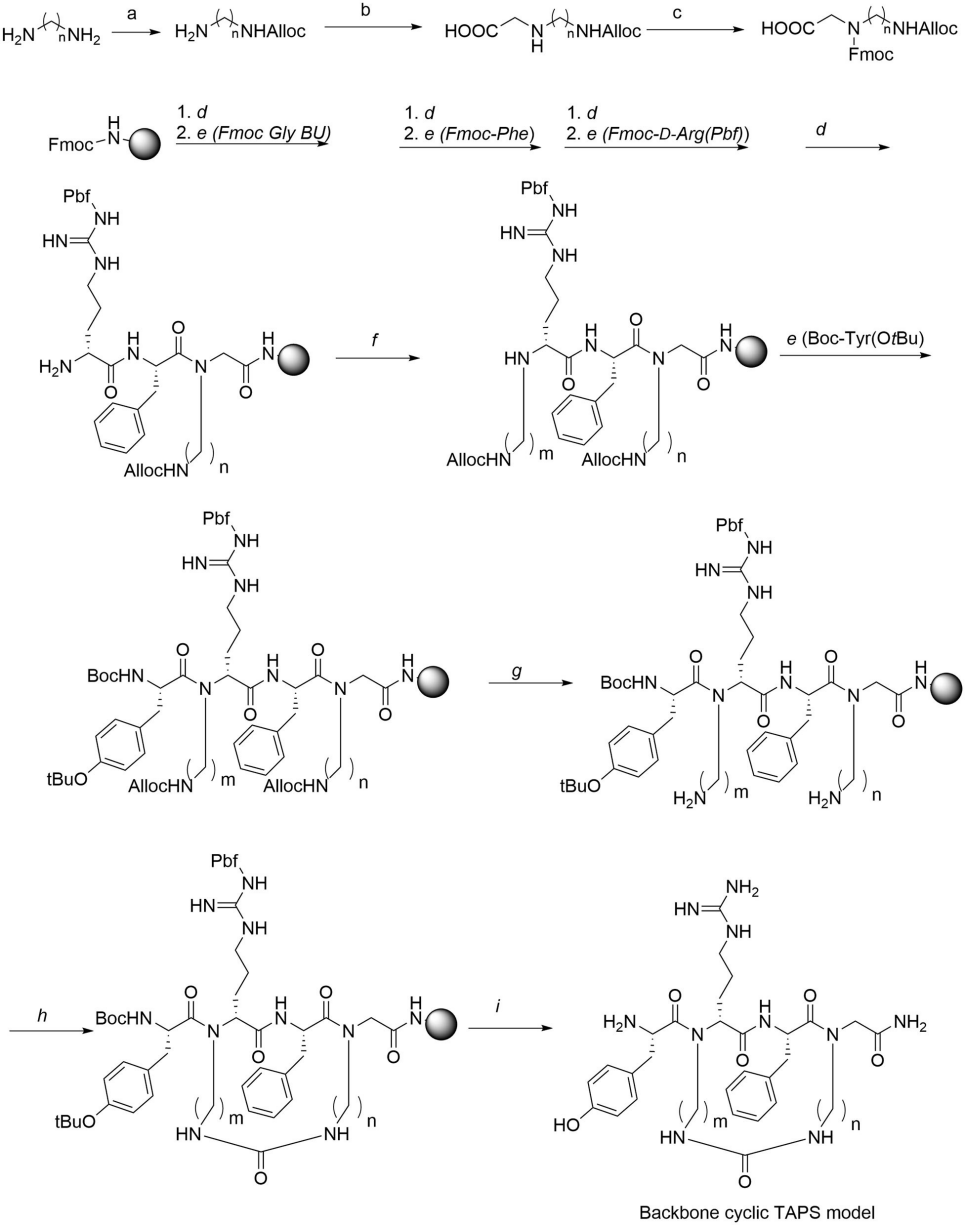
Synthesis of Gly-BU and TAPS c(n-m) backbone-cyclic peptide library. Reagents and conditions. a. Alloc-Cl, DCM. b. Glyoxylic acid, Sodium cyanoborohydride. c. Fmoc-OSu, Et_3_N. d. 20% piperidine/DMF. e. AA, HATU, DIPEA, DMF. f. Alloc-aminoalkyl aldehyde, NMP/MeOH, NaCNBH_3_. g. Pd(PPh_3_)_4_, PhSiH, DCM. h. BTC, DIPEA, DCM. i. TFA/TDW/TIS.

The synthesis of the TAPS c(n-m) library was performed on Rink amide MBHA resin using standard and modified Fmoc-SPPS protocols (Figure 5). The synthesis of the TAPS c(n-m) analogs posed two major challenges (Figure 5). The first challenge was coupling the sterically hindered *N*-alkylated amino acids to the preceding amino acid because standard conditions suffered from low conversion yields. This was overcome by using a more reactive coupling reagent 1-[Bis(dimethylamino)methylene]-1H-1,2,3-triazolo[4,5-b]pyridinium 3-oxide hexafluorophosphate, Hexafluorophosphate Azabenzotriazole Tetramethyl Uronium (abbreviated HATU), and optimized conditions (steps b, Figure 5) (Albericio and El-Faham, 2018). The second challenge was to improve the low yield of the *in-situ* reductive alkylation step on the solid support (step c, Figure 5). The reductive alkylation step proved to be most challenging because of the formation of the dialkylation by-product. This step resulted in low conversion yields under non-optimized conditions. We decided to go forward with the synthesis of the peptides without optimizing this step-in order to expedite the preparation of the library. This effort allowed us to obtain a sufficient amount of peptide for the initial biological screening (see section Scaling-Up the Synthesis of **TAPS c(2-6)** and Optimizing the On-Resin Reductive Alkylation Step below). To achieve the *N*-alkylation of Arg, we used the on-resin alkylation procedure instead of synthesizing Arg building units in advance. Hurevich and Gilon have developed a method for on-resin mono-alkylation of arginine using Alloc-protected alkyl aldehydes (Hurevich et al., 2007). In order to synthesis the focused library, Alloc-protected alkyl aldehydes with various alkyl chains were synthesized in a three-step procedure giving acceptable yields (Hurevich et al., 2007). Applying this method to the synthesis of the TAPS c(n-m) library enabled on-resin ring closure with a urea bond (step e, Figure 5), after removing the Alloc protecting groups (step d, Figure 5) using catalytic amounts of palladium (tetrakis). On-resin urea cyclization of the peptide (step e, Figure 5) was achieved according to the procedure described previously (Hurevich et al., 2007; Tal-Gan et al., 2011). Final peptide cleavage was performed under standard reaction conditions and peptides were purified by HPLC (see Experimental section) and characterized by MALDI-TOF MS (Table 1).

**Table 1:** Opioid receptor activity screening of cyclic TAPS analogs.

**Peptide**	**Ring size^**a**^**	**DOP** **agonist^**b**^** **(%)**	**DOP** **antagonist^**c**^** **(%)**	**MOP** agonist^d^^*^ **(%)**	**MOP** antagonist^e^^*^ **(%)**
**Linear**	–	14	10	90^**^	1
**TAPS** ***c*****(2-3)**	15	nd	2	24	1
**TAPS** ***c*****(2-6)**	18	11	6	48^**^	0
**TAPS** ***c*****(3-3)**	16	14	11	1	2
**TAPS** ***c*****(3-6)**	19	nd	1	1	2
**TAPS** ***c*****(4-3)**	17	11	0	63^**^	4
**TAPS** ***c*****(4-6)**	20	nd	3	1	1
**TAPS** ***c*****(6-3)**	19	19	8	75^**^	0
**TAPS** ***c*****(6-6)**	22	1	6	21	3

a *Ring size was determined by including the backbone and bridge atoms*.

b *At 10^−6^ M*.

c *Between 10^−9^-10^−6^ M*.

d *At 10^−6^ M*.

e *Between 10^−9^-10^−6^ M*.

* *All values lower than 25% are not significant*.

** *Significance p ≤ 0.01 compared to control*.

*nd, not determined*.

### *In vitro* Studies of TAPS c(n-m) Peptides

For the selection of a lead for further studies, the TAPS c(n-m) library was screened *in vitro* using the following three assays: (1) μ (MOP, OPRM1) and δ (DOP, OPRD2) opioid receptor functional agonism and antagonism, (2) metabolic stability, and (3) intestinal permeability using the Caco2 assay.

#### In-Cell Opioid Receptor Functional Assays of the TAPS c(n-m) Peptides

Peptides were tested for μ (MOP, OPRM1) and δ (DOP, OPRD2) opioid receptor activation functional agonism and antagonism using CHO cells expressing human recombinant μ receptors and NG-10815 cells which express significant endogenous levels of δ opioid receptors. The cultures were incubated for 10 min at 37°C followed by impedance measurements of CHO cells and cAMP was assayed by the HTRF method using NG-10815 cells, according to CEREP standard assay protocols. The respective reference, non-peptide small molecules were tested concurrently with the TAPS analogs. The agonist compounds were: DPDPE (selective agonist of DOP receptor) and DAMGO (high MOP receptor specificity) and the antagonist compounds were: Naltrindole (selective DOP receptor antagonist) and CTOP (MOP receptor antagonist). Table 1 displays the agonism and antagonism activity toward DOP and MOP receptors of representative members of the TAPS library. The results are expressed as a percentage of respective control-specific response. While the peptide library did not show significant functional agonist or antagonist activity for the DOP receptor, three compounds, **TAPS c(2-6)**, **TAPS c(4-3)** and **TAPS c(6-3)**, exhibited significant MOP agonist activation (48 ± 3, 63 ± 5, and 75 ± 9%, respectively) but no antagonist activity. These findings indicate that TAPS cyclic peptides are MOP selective full agonists. These peptide ring sizes include 17–19 atoms, indicating that specific ring size is required to allow binding to the MOP receptor and mimic the bioactive conformation of the linear TAPS. The cyclic TAPS peptide had lower activity than the linear parent TAPS peptide (90 ± 5%) – a finding that can be attributed to their restricted conformation relative to the flexible linear peptide conformation.

#### Stability Studies of TAPS Peptide

**Figure 6:**
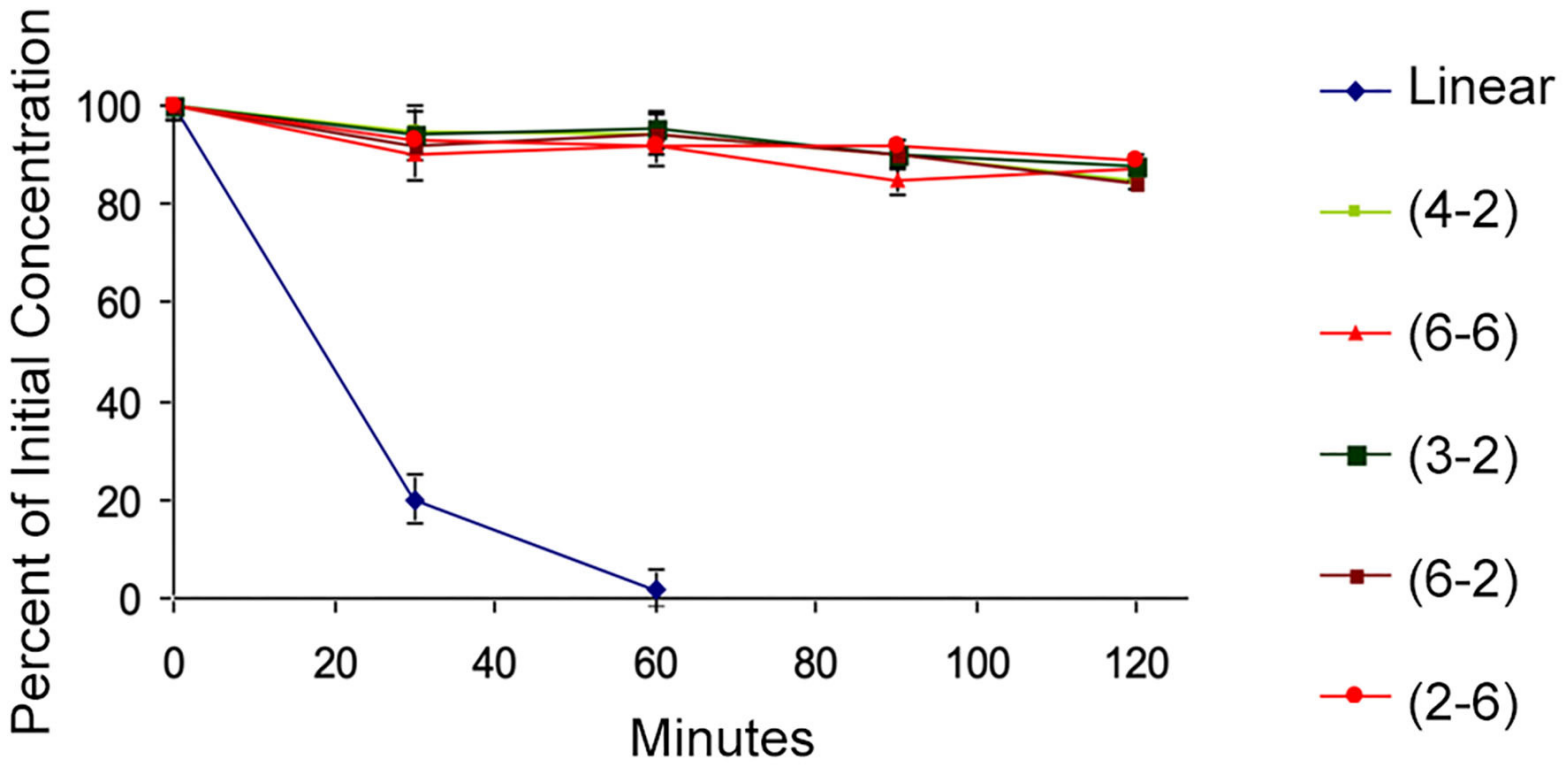
BBMV stability test. The cyclic peptides (high values, various colors) show stability to intestinal enzyme degradation compared to the linear analog (blue).

A few of the peptides from the library were selected for further evaluation. The **TAPS c(2-6)** representative MOP agonists, **TAPS c(6-2)** and **TAPS c(4-2)** were not evaluated for opioid receptor interaction, and two non-active cyclic peptides, **TAPS c(3-2)** and **TAPS c(6-6)**, having a smaller and larger ring sizes, respectively. The backbone-cyclic peptide stability in the GI tract was evaluated using rat origin pooled Brush Border Membrane Vesicle (BBMV) enzymes and were compared to the stability of linear TAPS (Figure 6).

The results show that the concentration of all cyclic peptides remained almost unchanged during exposure to intestinal enzymatic mixture for up to 120 min. In contrast, over 80% of the linear parent peptide was degraded after treatment for 30 min with intestinal enzymes. After 60 min all the linear peptides were decomposed. Our study indicates that all backbone-cyclic TAPS analogs are more stable than the linear peptide toward enzymatic degradation. It proved that the ring size has no influence on the stability, which indicates that the *N*-alkylation and the cyclization are the major contributors to the stability. This shows that the potential of backbone-cyclic TAPS analogs as therapeutic candidates is higher than that of the linear one.

#### Assessment of the *in vitro* Permeability Mechanism of TAPS c(2-6)

**Figure 7:**
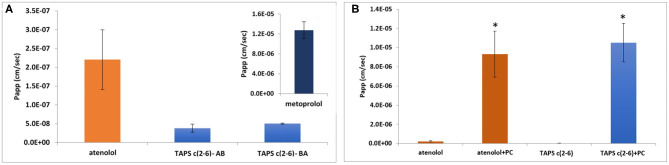
Permeability results in the Caco-2 permeability assay: assessment of directionality in the absorption of **TAPS c(2-6)** (60 μg/ml) across the epithelial monolayer. **(A)** AB vs. BA absorption of **TAPS c(2-6)** compared to the paracellular absorption marker, atenolol. (A insert) The Papp value of metoprolol, the marker for transcellular absorption. Values are presented as average ±SEM (*n* = 3). **(B)** Permeability results in the Caco-2 permeability assay. Papp values of **TAPS c(2-6)** (60 μg/ml) with and without the addition of PC (1 μg/ml) to the transport medium, compared to atenolol (10 μg/ml), the marker for paracellular diffusion. Values are presented as average ± SEM (*n* ≥ 6). *Significant difference (*p* < 0.05) from compound Papp without PC.

The permeability of the linear TAPS, the three active analogs **TAPS c(2-6)** and other conformers such as **TAPS c(6-2)** and **TAPS c(4-2)** were tested by the Caco-2 assay (Biron et al., 2008; Beck et al., 2012). The Caco-2 cells form an epithelial monolayer that greatly resembles the intestinal monolayer which determines if a certain compound will be absorbed and to what extent. These studies indicated that both the linear and the cyclic TAPS peptides had low permeability rates through the human epithelial colorectal adenocarcinoma cells. Their permeability rates were lower than the atenolol value, which is used as a standard for paracellular permeability. **TAPS c(2-6)**, among the metabolically stable analogs, was chosen for further evaluation of its permeability mechanism. **TAPS c(2-6)** was investigated in the Caco-2 model, utilizing directionality studies, where the permeability coefficient (Papp) is measured from apical to basolateral (AB) and from basolateral to apical (BA) sides. A ratio between the values over than 2, indicates an efflux system or transporter involvement that actively transfers the compound across the monolayer. When both AB and BA Papp values are similar, the permeability mechanism is passive diffusion and when comparing the values to atenolol or metoprolol it can be determined whether the pathway is paracellular or transcellular, respectively. The Papp of **TAPS c(2-6)** was measured in the AB direction and compared to its Papp value in the opposite BA direction. As can be seen in Figure 7A, the Papp values obtained in the AB vs. the BA direction were similar (3.8 × 10^−8^ vs. 5 × 10^−8^ cm/s, respectively) and were much lower than the transcellular marker, metoprolol (1.3 × 10^−5^ cm/s) and lower also than the paracellular marker atenolol (2.2 × 10^−7^ cm/s).

The low Papp value of **TAPS c(2-6)** led us to the assumption that **TAPS c(2-6)** crosses the epithelial membrane via paracellular diffusion, between the cells, as opposed to the transcellular pathway. To confirm this assumption, palmitoyl carnitine (PC) was added to the medium of the cells before and during the Caco-2 experiment. PC is an absorption enhancer that widens the gaps between the cells by disrupting the tight junctional complexes between them, thus enhancing the paracellular diffusion of solutes (Hochman and Artursson, 1994). The results of this study are presented in Figure 7B. As expected, the Papp value of atenolol, which is known to permeate the epithelial bilayer via paracellular diffusion, was significantly increased compared to the Papp value without PC (9.3 × 10^−6^ vs. 2.2 × 10^−7^ cm/s, respectively). Similarly, the Papp of **TAPS c(2-6)** was significantly higher when PC was added to the transport medium compared to the Papp value without PC (1.1 × 10^−5^ vs. 3.8 × 10^−8^ cm/s, respectively). With low Papp values of **TAPS c(2-6)** in the Caco-2, it is most likely to predict that the peptide would barely penetrate the blood-brain barrier (BBB), if at all. The BBB is much more regulated and stricter in allowing compound absorption (Wilhelm and Krizbai, 2014). While **TAPS c(2-6)** has shown negligible permeability across Caco-2 cells, pointing to minimal intestinal permeability, this may introduce an advantage for **TAPS c(2-6)** as peripheral pain killer. Peripheral pain killers are extremely beneficial since they are devoid of centrally-mediated side effects, such as addiction and respiratory suppression as they do not enter the CNS (Machelska and Celik, 2018). For example, morphine-6-beta-glucuronide which does not pass the BBB was suggested as a pure peripheral opioid pain killer (Stein et al., 2001; Tegeder et al., 2003). Several peptides have been suggested as selective peripheral binders of opioid receptors (Dooley et al., 1998; Stein et al., 2001). It is suggested that increasing hydrophilicity of the active peptide can be used to suppress BBB penetration and serve as a strategy to convert CNS active opioid agonists into peripheral active pain killers (Stein et al., 2001; Tegeder et al., 2003; Bedini and Spampinato, 2018). We assume the arginine of **TAPS c(2-6)** increases the hydrophilicity of the peptide compared to other peptide opioids e.g., the proline of endomorphine-2. **TAPS c(2-6)** is a pure MOP agonist and has low intestine permeability. This is an indication that it will not pass the BBB and might have selective peripheral opioid analgesia activity. Such a drug will be devoid of centrally-mediated side effects, such as addiction and respiratory suppression. We have pursued this line of development of **TAPS c(2-6)** as a peripheral pain killer devoid of morphine like side effects. These and additional results [e.g., PK and the effect of **TAPS c(2-6)** on respiratory arrest and addiction] will be published elsewhere in the future.

### Scaling-Up the Synthesis of TAPS c(2-6) and Optimizing the On-Resin Reductive Alkylation Step

**Figure 8:**
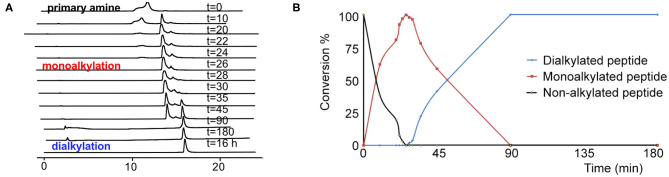
The kinetic pattern of the reductive alkylation of arginine with Alloc-6-aminohexanal. **(A)** HPLC time course of the reductive alkylation step on Arginine-peptidyl-resin using Alloc-6-aminohexanal. **(B)** The graph shows that reaction times of <20 min result in incomplete conversion. Prolonged reaction times, over 30 min, lead to a significant increase in the dialkylated side product.

Based on the combination of the three *in vitro* assays we concluded that **TAPS c(2-6)** showed improved stability in addition to its MOP receptor agonist activity. In order to evaluate the *in vivo* activity of **TAPS c(2-6)** we decided to scale-up its synthesis. While the non-optimized strategy enabled the synthesis of sufficient quantities of peptide to allow for initial screening, this strategy was not suitable for the preparation of large amounts due to the significant decrease in yield that was attributed to the non-optimized reductive alkylation step. Many attempts to form secondary amines from primary amines by using reductive alkylation resulted in low yields of the desired monoalkylated product and a significant amount of the undesired dialkylated product. Optimizing the conditions for the on-resin reductive alkylation step is crucial for the efficient and reproducible synthesis of backbone-cyclic peptides with non-glycine building blocks. Reductive alkylation on solid support is notorious for being non optimal which results in the formation of a mixture of non-, mono- or dialkylated product. While using short reaction times and low concentrations of aldehydes might result in incomplete alkylation, the use of high concentrations and longer reaction times can produce a predominantly dialkylated by-product. In both cases this causes a decrease in the yield and purity of the target compound. In order to enable more advanced studies of the **TAPS c(2-6)**, the overall synthetic strategy, and specifically the reductive alkylation step, were optimized to allow for larger scale synthesis. We figured that the most crucial synthetic step was the on-resin reductive alkylation of the Arg α-nitrogen. The step was challenging because the conversion of the primary amine to the monoalkylated secondary amine was accompanied by the formation of the undesired dialkylated by-product. Once the monoalkylated product is formed, a successive reaction with another aldehyde in the solution might take place to produce a second alkylation of the same amine. This implies that the ratio between the monoalkylated product and the dialkylated by-product might be time dependent. We decided to optimize the reductive alkylation step by evaluating the time required to get maximal conversion from the starting material to the monoalkylation product while minimizing the conversion to the dialkylated by-product. Arg-Phe-GlyBU2 peptides attached to the solid support were incubated with a mixture of three equivalents of Alloc-6-aminohexanal and NaCNBH_3_ for different time periods that ranged from 10 to 180 min. After each reaction, peptides were cleaved from the support (using small cleavage, see Supplementary Material) and the ratio between the non-alkylated, monoalkylated and dialkylated peptides was determined by HPLC (Figure 8). Our results showed that after 10 min only 60% conversion to the monoalkylated product was achieved and that even after 20 min there was still 20% of the non-alkylated reactant. We observed that if we left the reaction for 30 min the non-alkylated product was not present anymore but the dialkylated by-product started to form. Leaving the reaction for longer than 30 min showed a gradual increase in the formation of the dialkylated product. After 90 min only the dialkylated by-product was observed. These results indicated that there is a short time window in which the monoalkylation is dominant and the dialkylation is not yet significant. We evaluated the conversions in the time window between 20 and 30 min at a resolution of 2 min. After exactly 26 min the conversion to the monoalkylated product was maximal and the formation of the dialkylated product was minimal. The optimal conditions for on-resin reductive alkylation for this system were 26 min reaction time at room temperature using a mixture of three equivalents of aldehyde and reducing agent. The optimization of the alkylation step was used to prepare large amount of the **TAPS c(2-6)**. This allowed for NMR studies, elucidating the structure of **TAPS c(2-6)**, and for performing *in vivo* studies on animal models. The analytical evaluation studies performed here showed that it is possible to determine an accurate time window in which reductive monoalkylation is optimal. The exact conditions found here might be relevant only for the alkylation of Arg in **TAPS c(2-6)** model, however, the ability to accurately determine these conditions proved crucial for up-scaling the synthesis. The optimized reductive alkylation conditions for other peptides models could be substantially different and will require case-by-case optimization. It might be that optimizing such conditions for each peptide in a backbone-cyclic peptide library is not essential, but finding these conditions is very valuable for up-scaling the synthesis of selected backbone-cyclic peptides.

### *In vivo* Pharmacodynamic Studies of TAPS c(2-6)

#### Analgesic Effect—the Mouse Tail Flick Test

**Figure 9:**
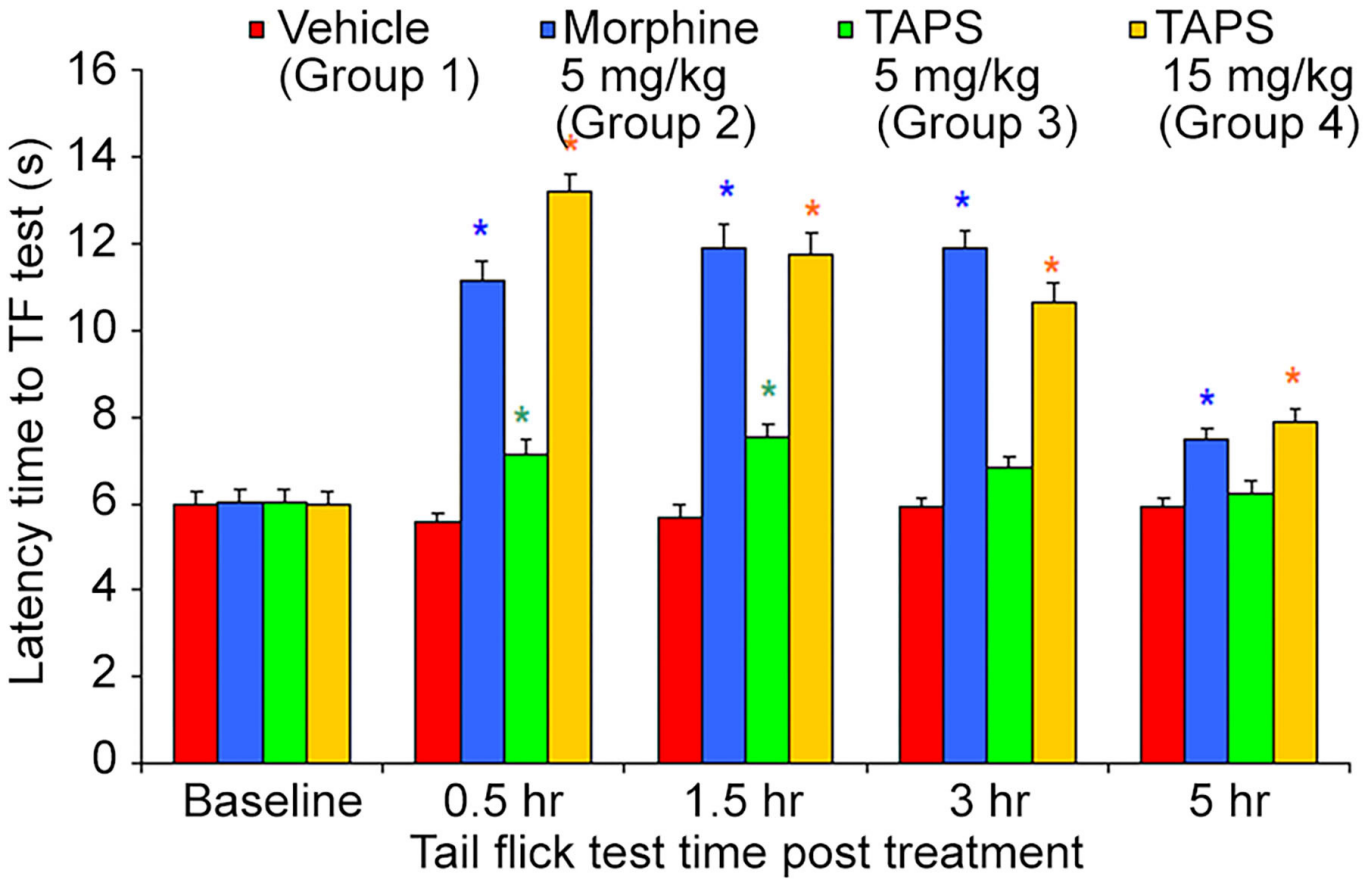
Latency time to tail flick of mice following IP administration of **TAPS c(2-6)** (5 or 15 mg/kg), morphine (5 mg/kg), or vehicle (PBS). Values are presented as mean ± SEM. *Significant difference (*p* < 0.05) from vehicle.

Following the preliminary results showing the *in vitro* agonist effect of **TAPS c(2-6)** in activating the μ-opioid receptor, the activity of **TAPS c(2-6)** in inducing analgesia *in vivo* was studied utilizing the mouse tail flick model. In this model, the mouse is exposed to local pain induced by an infrared light beam at the tail of the animal, and the time it takes for the animal to react to the pain, i.e., flick its tail, termed the “Latency time to tail flick,” is recorded. The treatment groups received either 5 or 15 mg/kg of **TAPS c(2-6)** dissolved in PBS. These were compared to animals receiving morphine (5 mg/kg) as a positive control or vehicle (PBS) as a negative control (Figure 9). The studies showed that both dosages of **TAPS c(2-6)** induced analgesia when compared to PBS. The analgesia induced by the higher dose, 15 mg/kg, was comparative in strength and duration to that induced by morphine.

**Figure 10:**
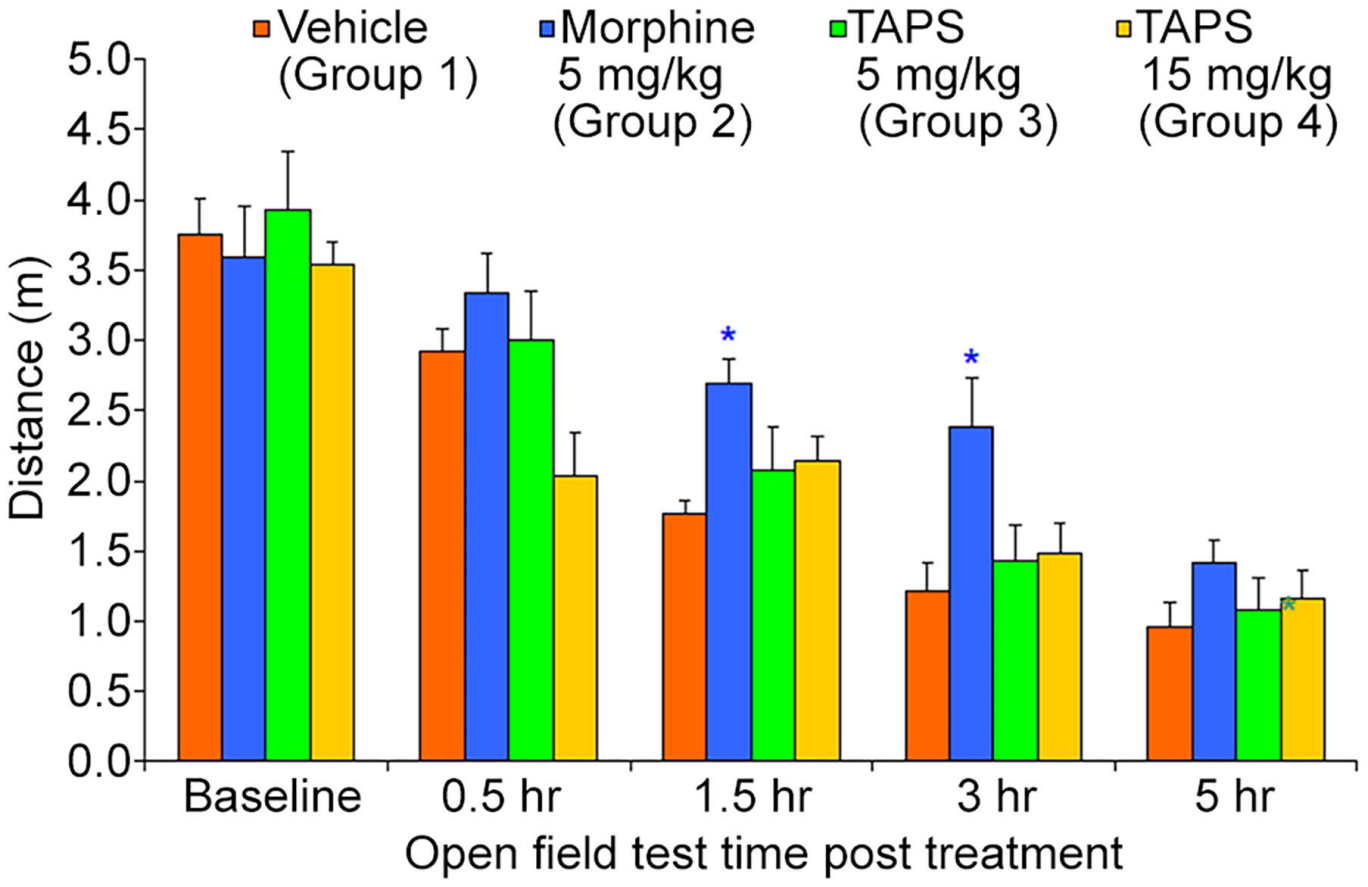
Distance of movement of mice over a 5-min period in the open-field apparatus following IP administration of **TAPS c(2-6)** (5 or 15 mg/kg), morphine (5 mg/kg), or vehicle (PBS). Values are presented as mean ± SEM. *Significant difference (*p* < 0.05) from vehicle.

**Figure 11:**
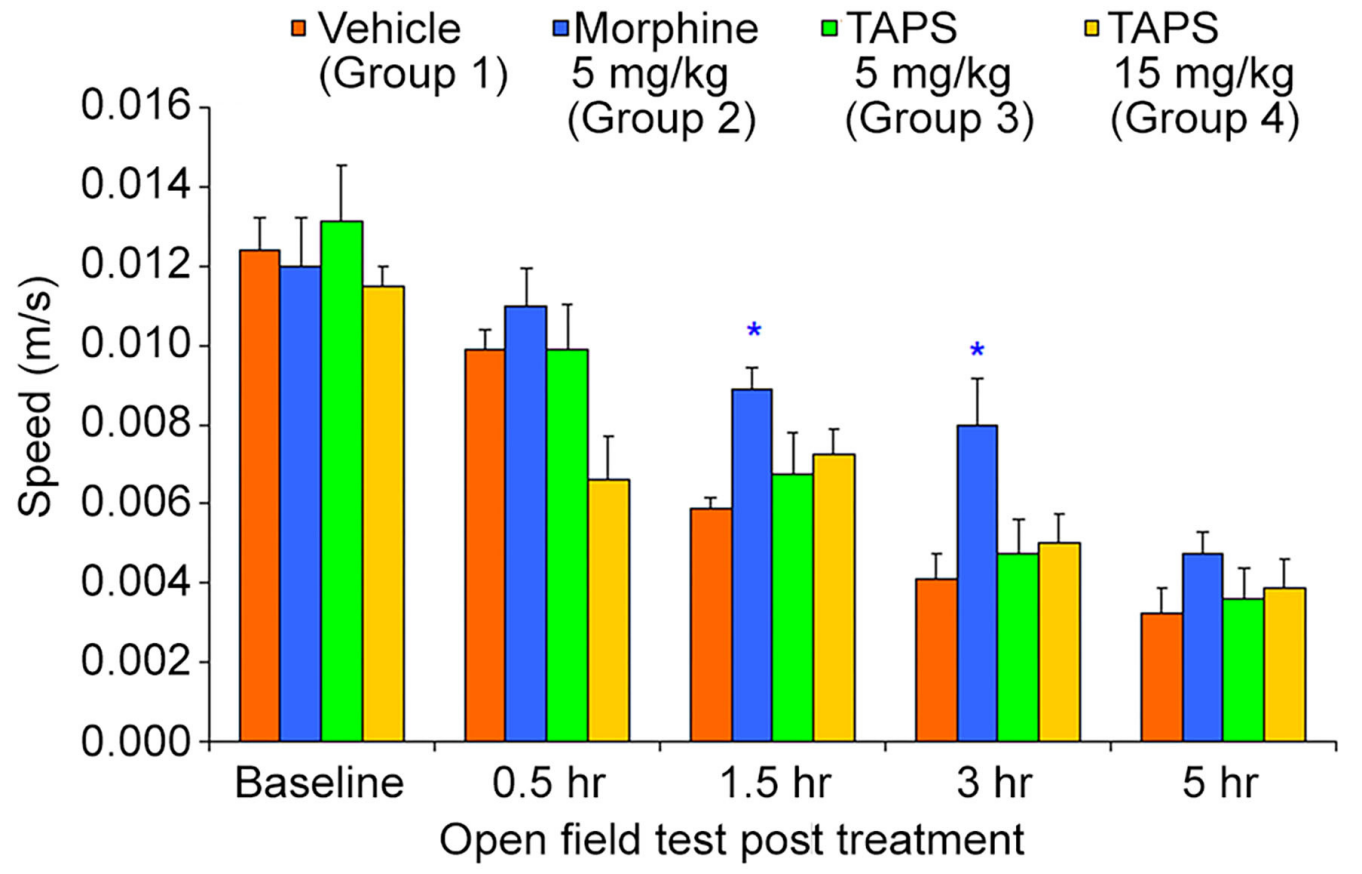
Speed of movement of mice over a 5-min period in the open-field apparatus following IP administration of **TAPS c(2-6)** (5 or 15 mg/kg), morphine (5 mg/kg), or vehicle (PBS). Values are presented as mean ± SEM. *Significant difference (*p* < 0.05) from vehicle.

Opioid analgesics are known for the side-effects they induce e.g., nausea, constipation, dizziness, and drowsiness. In order to assess the potential adverse effects **TAPS c(2-6)** might induce, we chose to examine its locomotor effect, which will give an indication to whether or not it affects the central nervous system as other opioids do. In the mouse open field test (OF test), the distance and speed the animal moves over a 5-min time period is recorded, giving an indication as to whether a medication administered affected its locomotor activity. **TAPS c(2-6)** (5 or 15 mg/kg) was administered via IP administration and compared to morphine (5 mg/kg) and vehicle (PBS). As expected, morphine induced an excitatory effect on the animals, causing the distance (Figure 10) and speed (Figure 11) of their movement in the OF apparatus to rise. In contrast, no locomotor effect was observed after administration of either dosage of **TAPS c(2-6)**.

Present pharmacodynamic studies were based on the characterization of **TAPS c(2-6)** as a full agonist of the MOP receptor. However, activity *in vitro* does not necessarily predict activity when administered *in vivo*. We sought to see whether **TAPS c(2-6)** was effective in inducing analgesia *in vivo*. The pharmacodynamics of **TAPS c(2-6)** involved both testing the peptide's analgesic activity *in vivo* and examining the possible side-effect profile. A dosage of 15 mg/kg **TAPS c(2-6)** induced analgesia *in vivo* similar in strength to that of morphine (5 mg/kg). Analgesia was also seen at a lower dosage of 5 mg/kg of **TAPS c(2-6)**. Most surprisingly, the analgesia induced at the higher dosage lasted for at least 5 h. The results from the *in vivo* studies are in line with the activity and stability studies *in vitro*. Both studies indicate that the stability of the cyclic peptide compared to the linear one contributes to the prolonged activity observed. While the activity of the cyclic peptide lead *in vivo* is comparable to that of morphine, the side effects were much subtler. This suggest that the cyclization can maintain the specific activity while preventing undesired ones. This advantage is a direct result of cyclization which enabled enough flexibility to bind the active site but also impeded conformations that led to unspecific activity.

### NMR Structural Analysis of the TAPS c(2-6)

**Figure 12:**
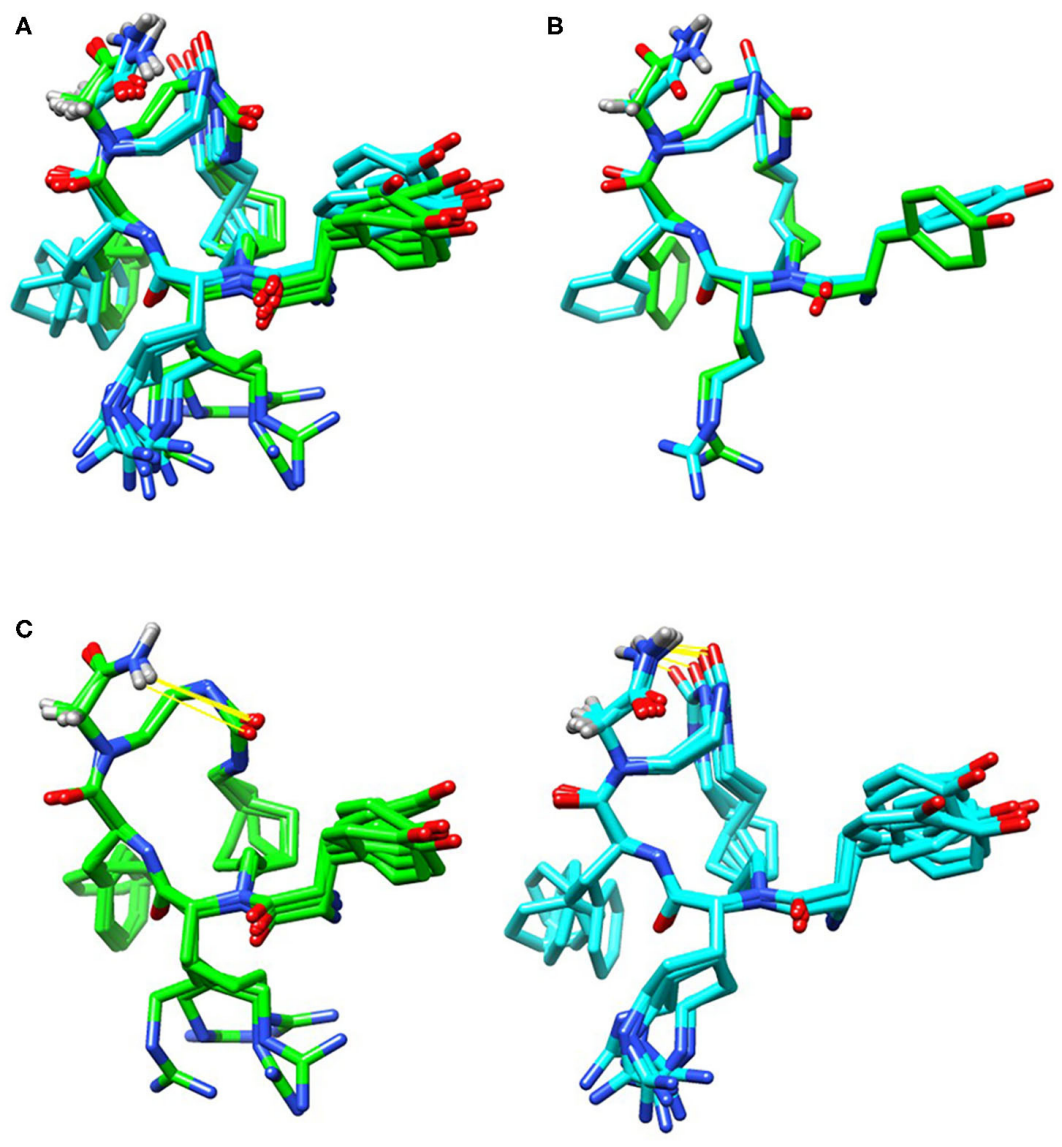
Low energy conformational ensembles of **TAPS c(2-6)** showing the entire ensemble colored by the lower energy (green) and higher energy (cyan) conformation ensembles **(A)**. These two groups differed in the relative orientation of the C-terminal amide and urea carbonyl **(B)**. Both conformations had hydrogen-bonds (in yellow) between the C-terminal amide and urea carbonyl **(C)**.

2D COSY, TOCSY, and ROESY spectra were used to assign resonances to all hydrogens in **TAPS c(2-6**) (see Supplementary Material for experimental details, Supplementary Table 1 for assignment and ^3^*J*_HNHα_ coupling values, and Supplementary Figure 1 for 1D and 2D superimposed spectra). The initial ensemble had a heavy atom RMSD of 2.92 Å, an RMSD of all C, N and Cα atoms of 1.09 Å and an RMSD of peptide backbone and heavy atoms of 0.48 and 1.47 Å, respectively. The lowest energy ensemble included 12 conformations with peptide backbone and heavy atom RMSD values of 0.21 and 0.98 Å, respectively. The urea carbonyl and C-terminus amide were hydrogen bound in all structures (Figure 12), however the conformations divided into two groups that differed in the directionality of the urea carbonyl relative to the plane of the ring. This semi-rigid structure can be required for the stability toward degradation and the long-term biological effect while allowing the peptide to adapt the active conformation of the linear active parent peptide.

The distances and relative orientation of the pharmacophoric moieties in the peptide, Tyr1, Phe4 and the N-terminus amine, were within the range described for peptides with a single bend (Deschamps et al., 1998) where the distance of the Phe ring to the N-terminal amine was 7.8 Å, and the Phe and Tyr rings were 8.3 Å apart; The relative angle of 119° was more characteristic of d-agonists. The ^3^*J*_HNHα_ values were consistent with the turn structure (Supplementary Table 1).

**Figure 13:**
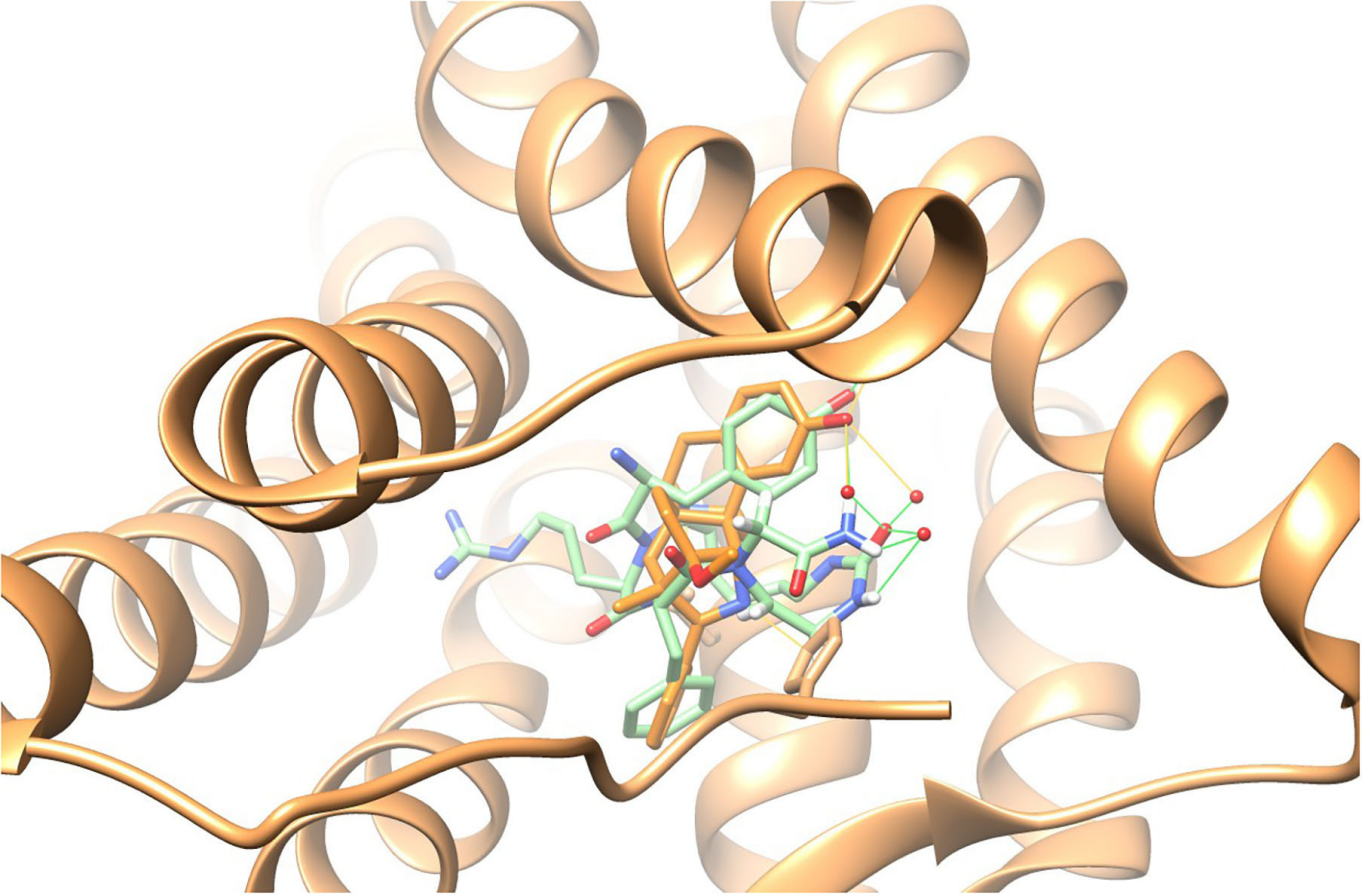
BU72 bound to the murine μ-opioid receptor determined by X-ray in orange (Huang et al., 2015) and the lowest energy structure of the **TAPS c(2-6)** ensemble (in green) superimposed on the pharmacophoric moieties at 1.2Å RMSD, showing similar positioning of the two aromatic rings and the N-terminal amide.

Previous studies have shown that the solution conformation of small, constrained cyclic peptides preserve and represent the native bioactive conformation (Marelli et al., 2014). The **TAPS c(2-6)** low energy conformation was compared to the morphinan agonist BU72 bound to the murine μ-opioid receptor determined by X-ray (Huang et al., 2015). The heavy atoms of the pharmacophoric moieties, Tyr, Phe and the N-terminal amine of **TAPS c(2-6)** could be superimposed onto those of BU72 within the Chimera program (see Supplementary Material) with a 1.2 Å RMSD. When superimposed onto BU72 within the solved structure of BU72 in the murine μ-opioid receptor, (PDB ID 5CM1, Huang et al., 2015), **TAPs c(2-6)** could form seven hydrogen bonds within the site, compared to eight hydrogen bonds formed by BU72 itself (Figure 13).

## Materials and Methods

### General Procedures for SPPS of TAPS Analogs

All peptides were synthesized on 0.1 g of Rink Amide MBHA resin scale (loading 0.7 mmol/g). The first three amino acids were introduced following microwave assistance SPPS protocols using discovery manual reactor (CEM). Coupling steps were performed by addition DIPEA (eight equiv) and HATU (three equiv) to a pre-cooled solution of Fmoc-Nα-XX-OH (three equiv.) in 8 mL NMP. The solution mixture was vortexed for 3 min and added to addition to the microwave reactor. Irradiated at 50 W for 10 min at 75°C. The peptidyl-resin was drained and the coupling reaction was repeated. The resin was washed with NMP. Fmoc protecting group was removed by irradiating of peptidyl-resin in a solution of 20% solution of piperidine in NMP at 50 W for 10 min at 75°C. After draining, the reaction was repeated, the peptide resin drained and washed with NMP. Reductive alkylation was performed in a vessel/shaker. The peptidyl-resin was washed three times with a mixture of NMP/MeOH (50/50 v/v) containing 1% AcOH. To the washed resin a solution of 1.9 equiv Alloc amino alkyl aldehyde in NMP/MeOH (50/50 v/v) 1% AcOH, under inert environment (argon) was added and sodium cyanoborohydride (0.025 g) were added, and the resin shaken for 30 min. Small cleavage was performed in order to make sure that the reaction gave the mono substituted product and the starting material was consumed. The resin was washed with NMP. After the reductive alkylation, Boc-Tyr(O-tBu)-OH was coupled by adding the resin to a pre-activated mixture of four equiv Boc-Tyr(O-tBu)-OH, four equiv HATU, 10 equiv DIEA in NMP. The vessel was shaken for 1 h at room temperature and the reaction repeated with new mixture after draining. Removal of Alloc protecting group was performed in vessel/shaker under inert conditions (argon) and in the dark (aluminum foil). 0.025 g of Pd(0) in a 20 mL mixture of 92.5% DCM, 5% AcOH and 2.5% NMM (N-Methylmorpholine) were added to the resin and shaken for 2 h at room temperature. Solution was drained and the resin was washed extensively using solution of: (1) 0.5% DIEA in DMF; (2) 0.025 g of sodium diethyldithiocarbamic acid trihydrate in 5 ml of DMF and NMP. Urea bridge cyclization was performed in vessel/shaker. The resin was washed with DCM, drained and followed by addition of 0.33 equiv of BTC in 25 mL DCM and the resin was shaken for 2 h. DIEA (8 equiv) was added to the resin solution and the reaction were shaken for further 16 h. The resin was washed with NMP. Cleavage of peptides was performed by suspending the peptidyl resin in a 10 mL of pre-cooled solution (at 0°C) of TFA (95%), triple distilled water (TDW) (2.5%) and TIS (2.5%). The mixture was shaken for 30 min at room temperature. The resin beads were then filtered out and the TFA filtrate was fully evaporated by a stream of nitrogen. The remained crude peptide was triturated with diethyl ether which was removed by decantation. The dried residue was dissolved in ACN:water 50% and lyophilized. The cleaved peptides were purified on a Merck-Hitachi HPLC using a reverse-phase C18 preparative column with a gradient range between 10 and 50% of acetonitrile (ACN) in TDW. ESI mass spectroscopy and analytical HPLC were used to check the identity and verify the peptide purity. All peptides were lyophilized and stored at −20°C. HPLC analyses were performed on a Waters e2695 system equipped with a pump, 2489 UV/Vis variable wavelength detector recording, and a column. Chromatograms were recorded at 280 nm at room temperature with a flow rate of 1 mL/min. The mobile phase consisted of solution A: TDW (0.1% v/v TFA) and solution B: ACN (0.1% v/v TFA). The detailed HPLC gradient program is presented below. The collected fractions were analyzed by MS. To obtain analytical HPLC chromatograms of crude peptides, all samples were dissolved in TDW/ACN 1:1 mixture, filtered through a 0.45 μm PTFE filters and injected to a reversed phase analytical HPLC column of Waters (XSelect CSH 130 Å C18, 4.6 × 150 mm, 3.5 μm).

### *In-vitro* Biological Assay of TAPS Analogs Procedures

#### Evaluation of the Agonist Activity of Compounds at the Human μ Receptor in Transfected CHO Cells, Determined by Measuring Their Effects on cAMP modulation Using the HTRF Detection Method

The cells are suspended in HBSS buffer (Invitrogen) complemented with HEPES 20 mM (pH 7.4) and 500 μM IBMX, then distributed in microplates at a density of 7.103 cells/well and preincubated for 10 min at room temperature in the presence of one of the following: HBSS (basal control), the reference agonist at 1 μM (stimulated control), the reference agonist (EC50 determination) or the test compounds. Thereafter, the adenylyl cyclase activator NKH 477 is added at a final concentration of 1 M. Following 10 min incubation at 37°C, the cells are lysed and the fluorescence acceptor (D2-labeled cAMP) and fluorescence donor (anti-cAMP antibody labeled with europium cryptate) are added. After 60 min at room temperature, the fluorescence transfer is measured at lex = 337 nm and lem = 620 and 665 nm using a microplate reader (Rubystar, BMG). The cAMP concentration is determined by dividing the signal measured at 665 nm by that measured at 620 nm (ratio). The results are expressed as a percent of the control response to 1 μM DAMGO. The standard reference agonist is DAMGO, which is tested in each experiment at several concentrations to generate a concentration-response curve from which its EC50 value is calculated.

#### Evaluation of the Agonist Activity of Compounds at the Rat delta2 Receptor Endogenously Expressed in NG-10815 Cells, Determined by Measuring Their Effects on Impedance Modulation Using the CellKey (CDS) Detection Method

Cells are seeded onto 96-well plate coated with fibronectin at 2 × 10^5^ cells/well in HBSS buffer (Invitrogen) + 20 mM HEPES (Invitrogen) with 0.1% BSA and allowed to equilibrate for 60 min at 37°C before the start of the experiment. Plates are placed onto the system and measurements are made at a temperature of 37°C. Solutions are added simultaneously to all 6-wells using an integrated fluidics system: HBSS (basal control), reference agonist at 1 μM (stimulated control), reference agonist (EC50 determination) or the test compounds. Impedance measurements are monitored for 10 min after ligand addition. The standard reference agonist is DPDPE, which is tested in each experiment at several concentrations to generate a concentration-response curve from which its EC50 value is calculated.

### In Cell Opioid Receptor Functional Assays

TAPS analogs (10-7-10-9 M), were tested in cellular functional assays using CHO cells expressing human recombinant μ (MOP, OPRM1) receptors and NG-10815 cells originally formed by fusion of mouse N18TG2 neuroblastoma cells with rat C6-BU-1 glioma cells which express significant endogenous levels of δ (DOP, OPRD2) opioid receptors. The respective reference, non-peptide small molecules were tested concurrently with the TAPS analogs; Agonist compounds DPDPE ([D-Pen2,D-Pen5]enkephalin), selective agonist of DOP receptor at 2 × 10^−9^ M and DAMGO ([D-Ala2, N-MePhe4, Gly-ol] enkephalin), high MOP specificity at 2.5 × 10^−9^ M; Antagonist compounds: naltrindole 7-(cyclopropylmethyl)-6,7-dehydro-4,5α-epoxy-3,14-dihydroxy-6,7-2′,3′-indolomorphinan hydrochloride, selective DOP antagonist, 5 × 10^−10^ M and CTOP (D-Phe-Cys-Tyr-D-Trp-Orn-Thr-Pen-Thr-NH2), MOP antagonist at 3.4 × 10^−7^ M. Depending on the assay volume and solvent tolerance, the stock solutions were diluted to [100×], [333×] or [1,000×] in 100% DMEM solvent, then either added directly or further diluted to [10×] or [5×] in H_2_O or Hank's balanced salt solution (HBSS) before addition to the assay well (final solvent concentration kept constant). The cultures were incubated for 10 min at 37°C followed by impedance measurements using cellular dielectric spectroscopy of CHO cells and cAMP was assayed by homogeneous time resolved fluorescence (HTRF) method using NG-10815 cells, according to CEREP standard assay protocols (https://euroscreenfast.com/assays/opioid-orl1-camp-fast-0360c; https://euroscreenfast.com/assays/opioid-op1-delta-camp-fast-0379c). The results (mean of three experiments) are expressed as a percent of control specific agonist response [(measured TAPS specific response/control specific agonist response) × 100] and as a percent inhibition of control specific agonist response [100 – (measured TAPS specific response/control specific agonist response) × 100] obtained in the presence of the TAPS compounds. Results showing a stimulation or an inhibition lower than 25% were considered not significant and mostly attributable to variability of the signal around the control level. Results showing a stimulation or an inhibition higher than 50% were considered to represent significant effects of the TAPS compounds.

### *In vivo* TAPS c(2-6) Pharmacodynamic Studies

Pharmacodynamic (PD) studies were performed following approval of an application form submitted by MD Biosciences Ltd. to the Committee for Ethical Conduct in the Care and Use of Laboratory Animals and the study complied with all rules and regulations accordingly. Experiments were carried out in the facilities of MD Biosciences, Ltd. according to the standard protocols of the company. Male ICR mice (Harlan, Israel) 7 weeks of age (25–27) were used for all pharmacodynamic experiments after 5-day acclimation to laboratory conditions. Animals were allowed free access to food (standard commercial, sterile rodent diet) and water throughout the experiments and kept in automatically controlled environmental conditions for temperature and humidity levels in a 12-h dark-light cycle.

### TAPS c(2-6) Analgesic Activity in the Mouse Tail-Flick Model

For the assessment of analgesia induced by **TAPS c(2-6)**, the mouse tail-flick model was used. Animals were randomly assigned to groups, six animals per group, receiving either **TAPS c(2-6)** (5 or 15 mg/kg solution in PBS), morphine (5 mg/kg) as positive control or PBS as vehicle, all administered via IP injection. Animals were placed upon the TF instrument surface (Ugo Basile S.R.L) and held in place. The tail was placed straight back over an infrared light source. The heat source and timer were turned on and automatically switched off when the mouse flicked its tail away from the heat source. The time it took for the animal to flick its tail was measured and results were recorded as latency time to TF. Baseline TF measurements were taken 1 day before initiation of the experiments and again at 0.5, 1.5, 3, and 5 h post injection. Prior to TF measurement, assessment of possible locomotor side-effects via the Open-Field model was performed, as will be described in the next section **TAPS c(2-6)** Adverse Effect Assessment- the Open-Field Test.

### TAPS c(2-6) Adverse Effect Assessment- the Open-Field Test

For assessment of possible locomotor adverse effects **TAPS c(2-6)** may induce, the open-field (OF) test was performed on the mice prior to TF testing. Animals were placed in the OF apparatus for a period of 5 min. The apparatus utilizes computerized monitoring which measures the speed and distance the animal walks during the measurement period. **TAPS c(2-6)** (5 or 15 mg/kg solution in PBS), Morphine (5 mg/kg) as positive control or PBS as vehicle were administered via IP injection to animals as described in the previous section **TAPS c(2-6)** Analgesic Activity in the Mouse Tail-Flick Model. Baseline measurements were taken 1 day before initiation of the experiments and again at 0.5, 1.5, 3, and 5 h post injection.

## Conclusions

This article demonstrates the applicability of our general approach of backbone cyclization and cycloscan for converting a linear peptide into a backbone-cyclic peptide lead with drug-like properties. This approach was applied to the pain reliever tetra peptide TAPS. In the design step, based on SAR studies, the bridge was anchored to the α nitrogens of Arg and Sar. We designed and synthesized a focused library with ring size diversity [called the TAPS c(n-m) library]. A combined set of biological assays were performed to screen the TAPS c(n-m) library for the selection of a potential drug-lead. The binding assay showed that some of the peptides were completely inactive, highlighting that peptides with ring sizes of 17–19 atoms were potential leads. The BBMV stability test indicated that all TAPS c(n-m) peptides had superior stability compared to the linear TAPS. We found that the combined activity and metabolic stability properties of **TAPS c(2-6)** were superior to the other members of the library and selected this peptide as a case study for further development as a drug-lead.

Our study highlighted that the reductive alkylation step is the bottleneck in the SPPS of **TAPS c(2-6)**. We showed that this on-resin step could be optimized by adjusting the reaction solvents and time to enable high-yield scale-up. The scale-up procedure used here enabled a more extensive *in vitro* and *in vivo* evaluation in animals. The preliminary *in vivo* studies indicated that **TAPS c(2-6)** offers an attractive alternative to the current treatment given today as it has similar *in vivo* effects as morphine. Moreover, Caco-2 permeability studies showed that **TAPS c(2-6)** has very low paracellular permeability. We concluded that **TAPS c(2-6)** would have low intestinal and BBB permeability and may be a lead for the development of a peripheral pain killer devoid of central adverse effects. The solution NMR studies showed that **TAPS c(2-6)** has two semi-rigid low energy conformations, which are stabilized by a hydrogen bond between the urea bridge and the terminal amide. The low-energy ensemble positions the pharmacophores in a comparable geometry as those of the morphine agonist BU72 as determined when bound to the murine μ-opioid receptor (Huang et al., 2015). The pharmacophores had relative internal orientations that were consistent with other opioids (Deschamps et al., 1998), all suggesting that a relatively rigid molecule has been formed that positions the necessary pharmacophoric elements in the correct positions for biologic activity.

This work demonstrates that backbone cyclization by on-resin *N*-alkylation strategies is a useful tool for developing an array of peptides with drug-like properties without interfering with the pharmacophores essential for bioactivity, while allowing the pharmacophores to be positioned in orientations required for binding. The resulting peptides are subsequently evaluated for pharmacologic activity. The implementation of this strategy to such a short peptide proved that it is especially valuable to preserve all the pharmacophore properties, in cases where other cyclization methods cannot be used.

## Data Availability Statement

All datasets generated for this study are included in the article/Supplementary Material.

## Ethics Statement

The animal study was reviewed and approved by Ethics Committee - research number: MD-17-15117-3.

## Author Contributions

AT, DR, AH, SH, and CG contributed conception and design of the study. MH, DES, AS, and CG organized the database. SH, OO, and SG performed the statistical analysis. AT and MH wrote the first draft of the manuscript. DR, SG, DES, AH, MH, AT, AS, PL, and CG wrote sections of the manuscript. All authors contributed to the article and approved the submitted version.

## Conflict of Interest

SH was employed by the company Meytav Technologies Incubator. The remaining authors declare that the research was conducted in the absence of any commercial or financial relationships that could be construed as a potential conflict of interest.
