# Supplementary material for: Cyclizing Painkillers: Development of Backbone-Cyclic TAPS Analogs
Source: Front Chem. 2020 Nov 12;8:532577. doi: 10.3389/fchem.2020.532577 (PMC7689096; doi:10.3389/fchem.2020.532577)
Supplement: Supplementary file 1 [file Data_Sheet_1.PDF]

Supplementary Material

Contents

1. Mass spectrometry analyses 1
2. Metabolic Stability in BBMVs 1
   2.1 Preparation of BBMVs 1
   2.2 Experimental protocol for BBMVs 2
3. Permeability 2
   3.1 In vitro cell based model: Caco-2 cell culture permeability studies 2
      3.1.1 Growth and Maintenance of the Cells: 2
      3.1.2 Preparation of the Cells for Transport Studies: 2
   3.2 Experimental Protocol 2
   3.3 Data Analysis of *In Vitro* Studies 3
   3.4 Statistical Analysis of the *In Vitro* Studies 3
   3.5 Statistical Analysis of Pharmacodynamic Studies 3
   3.6 Analytical Methods for Compound Concentration 3
4. NMR studies 5
5. Supplementary Figures 6
6. Supplementary Tables 7

1. Mass spectrometry analyses

Mass spectra were acquired on a LCQ Fleet Ion Trap mass spectrometer (Thermo Scientific) utilizing electrospray ionization. For high resolution mass spectrometry (HRMS) analyses, the spectra were recorded on an Agilent 6550 iFunnel Q-TOF LC/MS system.

2. Metabolic Stability in BBMVs

2.1 Preparation of BBMVs

Brush border membrane vesicles (BBMVs) were prepared from combined duodenum, jejunum, and upper ileum by a Ca^{2+} precipitation method.(Kessler et al., 1978; Peerce, 1997) The intestines of five male Wistar rats at a weight of 200–250 g were rinsed with ice cold 0.9% NaCl solution and freed of mucus; the mucosa was scraped off the luminal surface with glass slides, put immediately into a buffer containing 50 mM KCl and 10 mM Tris-HCl (pH 7.5, 4 °C) and then homogenized (Polytron PT 1200, Kinematica AG, Switzerland). $CaCl_2$ was added to a final concentration of 10 mM. The homogenate was left shaking for 30 min at 4 °C and then centrifuged at 10,000 g for 10 min. The supernatant was then centrifuged at 48,000 g for 30 min, and two additional purification steps were undertaken by suspending the pellet in 300 mM mannitol and 10 mM Hepes/Tris (pH 7.5) and centrifugation at

24,000g for 60 min. Purification of brush border membranes was assayed using the brush border membrane enzyme markers c-glutamyl transpeptidase (GGT), leucine amino peptidase (LAP), and alkaline phosphatase (ALP). During the course of these studies, enrichment in brush border membrane enzymes varied between 13- and 18-fold.

2.2 Experimental protocol for BBMVs

Stock solutions of the tested peptides were diluted in 50 mM MES buffer (pH 7.4) and mixed with purified BBMVs (2:1). Metoprolol (20 µg/ml) was added to the test solutions as an internal standard for the procedure. The test solutions were incubated in a water bath at 37 °C for 90 min. Triplicate samples (50 µL) were taken at predetermined time points (0, 15, 30, 45, 60, and 90 min). The enzymatic reaction was stopped by adding 200 µL of ice-cold ACN to the samples (tubes A) and the samples were centrifuged (10,000 g, 10 min). The supernatant was collected and transferred to fresh glass test tubes (tubes B) and evaporated to dryness (Vacuum Evaporation System, Labconco, Kansas City, MO). Then, tubes B were reconstituted in 100 µL of mobile phase (15:85 ACN:water with 0.1% formic Acid) and vortexed for 1 min. The reconstituted samples were further centrifuged (10,000 g, 10 min) to ensure purity of samples, transferred to HPLC vials and frozen pending analysis by HPLC-MS, as will be described.

3. Permeability

3.1 In vitro cell based model: Caco-2 cell culture permeability studies

3.1.1 Growth and Maintenance of the Cells:

Caco-2 cells were obtained from ATCC (Manassas, VA, USA) and grown in 75 cm^2 flasks with approximately 0.75×10^6 cells/flask at 37 °C in a 5% CO_2 atmosphere and at a relative humidity of 95%. The culture growth medium consisted of Dulbecco's Modified Eagle Medium supplemented with 10% heat-inactivated fetal bovine serum, 1% nonessential amino acids, 2 mM sodium pyruvate, 2 mM penicillin-streptomycin solution and 2 mM L-glutamine. The medium was replaced three times weekly.

3.1.2 Preparation of the Cells for Transport Studies:

For the transport studies, cells in a passage range of 53-60 were seeded at a density of 25×10^5 cells/cm^2 on pretreated culture inserts of a polycarbonate membrane with 0.4 µm pores and a surface area of 1.1 cm^2 and then placed in 12-well transwell plates, 12 mm, Costar™. The culture medium was changed every other day. Transport studies were performed 21-22 days after seeding, allowing the cells proper proliferation, differentiation and development of their proper morphology.

3.2 Experimental Protocol

Transport studies (apical to basolateral, A to B) were initiated by removing the medium from both sides of the monolayer and replacing it with 600 µL of apical buffer (0.025 M D-glucose monohydrate, 0.02 M MES biological Buffer, 1.25 mM calcium chloride and 0.5 mM magnesium chloride in Hanks Balanced Salt Solution, filtered and titrated to pH 6.5 with NaOH) and 1500 µL of basolateral buffer (0.025 M D-glucose monohydrate, 0.02 M HEPES biological buffer, 1.25 mM calcium chloride, and 0.5 mM magnesium chloride in Hanks Balanced Salt Solution, filtered and titrated to pH 7.4 with NaOH), both preheated to 37 °C. The cells were incubated for 30 min at 37 °C with shaking (80 cycles/min). After the incubation period, the buffers were removed and replaced with 1500 µL of basolateral buffer on the basolateral side. Test solutions containing TAPS c(n-m) library peptides (60 µg/ml) in apical buffer were preheated to 37 °C and added (600 µL) to the apical side of the monolayer. Samples (50 µL) were immediately taken from the apical side at the beginning of the experiment,

leaving a 550 µL apical volume during the experiment. For the period of the experiment, the cells were kept at 37 °C with shaking. At predetermined times (20, 40, 60, 80. 100, 120 and 150 min), 200 µL samples were taken from the basolateral side and replaced with the same volume of fresh basolateral buffer to maintain a constant volume and sink conditions. For the basolateral to apical study (B to A), the test solution of TAPS c(n-m) library peptides were placed in the basolateral chamber, followed by immediate sampling from the basolateral side and continued sampling from the apical side at predetermined times, similarly to the A to B protocol. Samples were kept frozen at a temperature of -20°C pending analysis by HPLC-MS, as will be described.

3.3 Data Analysis of *In Vitro* Studies

The samples obtained from the Caco-2 permeability experiments were analyzed for peptide, atenolol and metoprolol content using the HPLC-MS system, as will be described. The permeability coefficient (Papp) of each peptide was calculated from the linear plot of drug accumulated vs. time, using the following equation:

$$Papp = \frac{dQ/dt}{C_0 * A}$$

where dQ/dt is steady state appearance rate of the drug on the receiver side, C_0 is the initial concentration of the drug on the donor side, and A is the exposed tissue surface area, 1.1 cm^2 in the specified experiments.

3.4 Statistical Analysis of the *In Vitro* Studies

All values are expressed as mean ± standard error of the means (SEM) if not stated otherwise. To determine statistically significant differences among the experimental groups, the two-tailed paired student's *t*-test was used. A *p*-value of less than 0.05 was termed significant.

3.5 Statistical Analysis of Pharmacodynamic Studies

Data are presented as mean ± standard error of the mean (SEM). Data was analyzed using one-way ANOVA followed by Tukey post-test (GraphPad) comparing treatment groups receiving **TAPS c(2-6)** or morphine to the group treated with vehicle. A p value < 0.05 was considered to represent a significant difference.

3.6 Analytical Methods for Compound Concentration

After sample handling and extraction procedures in the various experiments, compound concentrations were analyzed using an HPLC system (Waters 2695 Separation Module) equipped with a Waters Micro-mass ZQ mass-spectrometer (Waters corporation, Milford, MA). Each compound was analyzed according to specified analytical procedures, as follows.

Atenolol 10 µL of the sample was injected into the HPLC-MS system conditioned as follows: Kinetex EVO C18, 2.6 µm, 100 A, 100 × 2.1 mm column (Phenomenex®, Torrance, CA), an isocratic mobile phase, ACN:water (15:85 v/v) supplemented with 0.1% formic acid, flow rate of 0.1 mL/min at 40 °C. Nitrogen flow was 500 L/hr, desolvation temperature was 350 °C, source temperature was 110 °C and the cone voltage was 22 V. The range of quantification for all the compounds was 10–0.05 µg/mL. Retention times were 3.9 and 6.5 min for atenolol and metoprolol, respectively. Detection masses (*m/z*) were 267.3 for atenolol and 268.3 for metoprolol.

TAPS c(2-6) 10 µL of the sample was injected into the HPLC-MS system conditioned as follows: Xterra MS C18 column (3.5 µm, 2.1 × 100 mm, Waters, Milford, MA, USA), an isocratic mobile phase, ACN:water (10:90 v/v) supplemented with 0.1% formic acid, flow rate of 0.1 mL/min at 35 °C. Nitrogen flow was 500 L/hr, desolvation temperature was 350 °C, source temperature was 110 °C and the cone voltage was 30 V. Retention time was 5.5 min. The range of quantification was 10–0.05 µg/mL. Detection mass (*m/z*) was 346.77.

4. NMR studies

TAPS c(2-6) sample (5.2 mg in 10% D2O/H2O) was dissolved from lyophilized form. NMR experiments were performed on a Bruker 500 MHz DRX spectrometer using a 5-mm selective probe equipped with a self-shielded xyz-gradient coil at 24.2 °C. The transmitter frequency was set on the water signal and calibrated at 4.81 ppm. Correlation spectroscopy (COSY) (Aue et al., 1976) total correlation spectroscopy (TOCSY) (Bax and Davis, 1985a) (Bax and Davis, 1985) and Rotating frame Overhauser Effect spectroscopy (ROESY) (Bax and Davis, 1985b) experiments were acquired under identical conditions. The TOCSY experiment used mixing times of 100 ms, the ROESY used a spin lock of 250 mS and both used gradients for water suppression (Liu et al., 1998). Spectra were processed and analyzed with TopSpin (Bruker Analytische Messtechnik GmbH) and NMRFAM-SPARKY programs (Lee et al., 2015). Resonance assignment was done according to the sequential assignment methodology developed by Wuthrich (Wüthrich, 1986). Peak intensities were manually assigned as strong (from the Van der Waals radius, 1.8 Å – 2.3 Å), medium (1.8 Å – 3.3 Å), weak (1.8 Å – 4.3 Å) and very weak (1.8 Å – 5.3 Å) from 2D homonuclear ROESY experiments (see Supplementary Tables 2 and 3 for NOE connectivity statistics and data). An ensemble of 50 structures were calculated by the hybrid distance geometry-dynamical simulated annealing method using XPLOR version 3.856 (Nilges et al., 1991). The bridge unit was introduced using patches within XPLOR with canonical atom and bond geometry. The NOE energy was introduced as a square-well potential with a force constant of 50 kcal/mol·Å2 that was constant throughout the protocol. Each round of simulated annealing refinement consisted of 300 1-fs steps at 3500 K and 20,000 1-fs steps during cooling to 300 K. Finally, the structures were minimized using conjugate gradient energy minimization for 1000 iterations. These structures were used to stereospecifically assign resolved peaks by comparing energies for both options for each resolved methylene and using the lower energy option. There were 50 initial structures. Low energy structures chosen for further analysis had no ROE violations, deviations from ideal bond lengths of less than 0.05 Å, and bond angle deviations from ideality of less than 5º. Structural analysis and figures were produced using Chimera (supported by NIH P41 RR-01081).(Pettersen et al., 2004) Superpositioning was performed using least-squares fitting of specified atoms and the least-squares-fit root-mean-square deviation (RMSD) was reported. Hydrogen bonds were determined by Chimera relaxing canonical values by 0.7 Å and 50°, taking the flexibility of the molecule into account.

5. Supplementary Figures

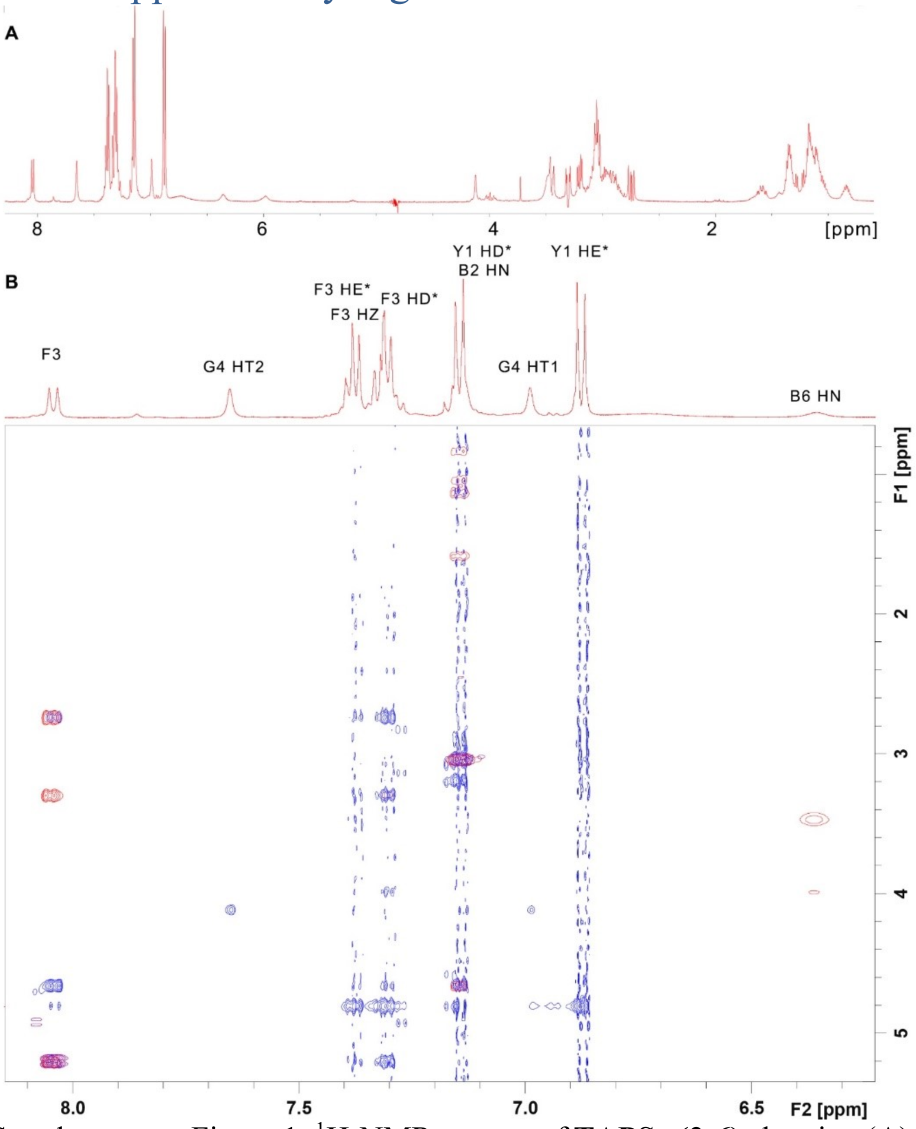

Supplementary Figure 1. ^{1}H-NMR spectra of **TAPS c(2-6)** showing (A) 1D spectrum and (B) fingerprint region of 2D spectrum showing ROESY (blue), TOCSY (red) and COSY(purple) superimposed spectra and the 1D spectrum from the corrosponding region with amino acid assignment on top.

6. Supplementary Tables

Supplementary Table 1. Assignment of hydrogens in NMR spectrum of **TAPS c(2-6)** and $^3J_{HNH\alpha}$ values for the amide protons. Bri2 and Bri6 are the ethyl and hexyl sides of the urea bridge, respectively, with numbering starting from the urea amide. HT is the amide at the C-terminus of Gly4.

	HN (ppm) ($^3J_{HNH\alpha}$, Hz)	Hα	Hβ	Hγ	Hδ	Hε	Hζ	HT
Tyr1		4.58	3.21, 3.08		7.16	6.87		
Arg2		3.02	1.35, 1.13	1.35, 1.13	2.91			
Phe3	8.04 (9.1)	5.21	3.30, 2.74		7.31	7.37	7.32	
Gly4		4.12						7.65, 6.98
Bri2	7.14 (8.5)	3.04	1.04, 0.84	1.12, 0.84		1.58	4.66	
Bri6	6.35 (broad)	3.45	3.99					

Supplementary Table 2: NOE connectivity data.

**NOE Connectivity**	
Total	54
i,i	36
i,i+1	10
i,i+2	3
Interactions peptide-bridge	9
**RMSD**	
Non-violated	50/50
C, N and Cα atoms (Å)	1.09
Heavy (Å)	2.92
Presented ensemble conformations	12/50
Backbone (Å)	0.21
Heavy (Å)	0.98

Supplementary Table 3. NOE connectivity data (PDB nomenclature).

**Amino acid 1**	**Proton type**	**Amino acid 2**	**Proton type**	**Interaction strength (s - strong, m - medium, w - weak, vw - very weak)**
**Phe3**	HA	Phe3	HN	vw
**Phe3**	HB2	Phe3	HN	m
**Phe3**	HD*	Phe3	HN	vw
**Bri6**	HA*	Bri6	HN	m
**dArg2**	HB*	Phe3	HN	vw
**Bri6**	HZ*	Phe3	HN	s
**Tyr1**	HB1	Bri2	HN	vw
**Tyr1**	HA	Tyr1	HB2	vw

**Tyr1**	HA	Tyr1	HB1	vw
**Tyr1**	HA	Tyr1	HD*	vw
**Tyr1**	HB2	Tyr1	HD*	vw
**Tyr1**	HB1	Tyr1	HD*	w
**Tyr1**	HE*	Tyr1	HD*	s
**dArg2**	HB*(Aue et al.)	dArg2	HA	m
**dArg2**	HB*(Aue et al.)	dArg2	HD*	m
**dArg2**	HB*(Aue et al.)	dArg2	HA	vw
**dArg2**	HB*(Aue et al.)	dArg2	HD*	s
**Phe3**	HA	Phe3	HB1	s
**Phe3**	HA	Phe3	HB2	m
**Phe3**	HA	Phe3	HD*	m
**Phe3**	HA	Phe3	HE*	vw
**Phe3**	HA	Phe3	HZ	vw
**Phe3**	HB1	Phe3	HA	w
**Phe3**	HB1	Phe3	HD*	m
**Phe3**	HB1	Phe3	HE*	vw
**Phe3**	HB2	Phe3	HA	vw
**Phe3**	HB2	Phe3	HB1	s
**Phe3**	HB2	Phe3	HD*	m
**Phe3**	HB2	Phe3	HE*	vw
**Phe3**	HB2	Phe3	HZ	w
**Gly4**	HA*	Gly4	HT1	w
**Gly4**	HA*	Gly4	HT2	w
**Bri6**	HB2	Bri6	HE*	vw
**Bri6**	HB1	Bri6	HE*	m

**Bri6**	HE*	Bri6	HA*	m
**Bri6**	HZ*	Bri6	HA*	m
**Bri6**	HZ*	Bri6	HE*	w
**Bri2**	HA*	Bri2	HB*	s
**Bri2**	HB*	Bri2	HA*	s
**Tyr1**	HA	dArg2	HB*G*	vw
**Tyr1**	HE*	dArg2	HB*G*	vw
**Phe3**	HA	dArg2	HB*G*	w
**Phe3**	HA	dArg2	HB*G*	vw
**Phe3**	HA	Gly4	HA*	vw
**Gly4**	HA*	Phe3	HB1	vw
**Bri6**	HZ*	Phe3	HD*	vw
**Bri2**	HA*	Gly4	HA*	m
**Phe3**	HA	Bri2	HB*	s
**Phe3**	HB1	Bri2	HB*	m
**Bri6**	HE*	dArg2	HD*	m
**Bri6**	HZ*	dArg2	HA	s
**Bri6**	HZ*	dArg2	HB*G*	m
**Bri2**	HB*	Phe3	HA	m
**Bri2**	HB*	Phe3	HB2	vw

Supplementary Table 4. Analytical HPLC program

Time (min)	% TDW	% ACN
**0**	95	5
**1**	95	5
**6**	78	22
**16**	68	32
**20**	5	95
**22**	5	95
**24**	95	5

Supplementary Table 5. Characterization of the TAPS c(n-m) library

Peptide	n	m	Ring Size[a]	MW (g/mol) (Calculated)	MW[b] (g/mol) (Observed)	HPLC[b] *k'* (min)
**TAPS**			Linear	555.28	554.70	5.45
**TAPS c(2-2)**	2	2	14	653.33	655.47	9.27
**TAPS c(3-2)**	3	2	15	667.34	669.35	9.35
**TAPS c(4-2)**	4	2	16	681.36	681.83	9.40

**TAPS c(6-2)**	6	2	18	709.39	710.55	9.43
**TAPS c(2-6)**	2	6	18	709.39	711.87	9.42
**TAPS c(3-6)**	3	6	19	723.41	724.69	9.51
**TAPS c(4-6)**	4	6	20	737.41	735.66	9.58
**TAPS c(6-6)**	6	6	22	765.45	769.20	9.70

[a] Ring size was determined by including all the atoms in the ring including the bridge and the backbone atoms. [b] For instrumentation and conditions see above.

Aue, W.P., Bartholdi, E., and Ernst, R.R. (1976). 2-Dimensional Spectroscopy - Application to Nuclear Magnetic-Resonance. *J Chem Phys* 64(5), 2229-2246. doi: Doi 10.1063/1.432450.

Bax, A., and Davis, D.G. (1985a). Mlev-17-Based Two-Dimensional Homonuclear Magnetization Transfer Spectroscopy. *J Magn Reson* 65(2), 355-360. doi: Doi 10.1016/0022-2364(85)90018-6.

Bax, A., and Davis, D.G. (1985b). Practical Aspects of Two-Dimensional Transverse Noe Spectroscopy. *J Magn Reson* 63(1), 207-213. doi: Doi 10.1016/0022-2364(85)90171-4.

Kessler, M., Acuto, O., Storelli, C., Murer, H., Muller, M., and Semenza, G. (1978). A modified procedure for the rapid preparation of efficiently transporting vesicles from small intestinal brush border membranes. Their use in investigating some properties of D-glucose and choline transport systems. *Biochim Biophys Acta* 506(1), 136-154. doi: 10.1016/0005-2736(78)90440-6.

Lee, W., Tonelli, M., and Markley, J.L. (2015). NMRFAM-SPARKY: enhanced software for biomolecular NMR spectroscopy. *Bioinformatics* 31(8), 1325-1327. doi: 10.1093/bioinformatics/btu830.

Liu, M.L., Mao, X.A., Ye, C.H., Huang, H., Nicholson, J.K., and Lindon, J.C. (1998). Improved WATERGATE pulse sequences for solvent suppression in NMR spectroscopy. *J Magn Reson* 132(1), 125-129. doi: DOI 10.1006/jmre.1998.1405.

Nilges, M., Kuszewski, J., and Brünger, A.T. (1991). "Sampling Properties of Simulated Annealing and Distance Geometry," in *Computational Aspects of the Study of Biological Macromolecules by Nuclear Magnetic Resonance Spectroscopy,* eds. J.C. Hoch, F.M. Poulsen & C. Redfield. (Boston, MA: Springer US), 451-455.

Peerce, B.E. (1997). Interaction of substrates with the intestinal brush border membrane Na/phosphate cotransporter. *Biochim Biophys Acta* 1323(1), 45-56. doi: 10.1016/s0005-2736(96)00174-5.

Pettersen, E.F., Goddard, T.D., Huang, C.C., Couch, G.S., Greenblatt, D.M., Meng, E.C., et al. (2004). UCSF chimera - A visualization system for exploratory research and analysis. *J Comput Chem* 25(13), 1605-1612. doi: 10.1002/jcc.20084.

Wüthrich, K. (1986). *NMR of Proteins and Nucleic Acids.* John Wiley & Sons, Inc., New York.
